# Penilloic acid is the chief culprit involved in non-IgE mediated, immediate penicillin-induced hypersensitivity reactions in mice

**DOI:** 10.3389/fphar.2022.874486

**Published:** 2022-08-22

**Authors:** Dunfang Wang, Jiayin Han, Chen Pan, Chunying Li, Yong Zhao, Suyan Liu, Yushi Zhang, Jingzhuo Tian, Yan Yi, Jingjing Zhu, Chenyue Liu, Yuan Wang, Zhong Xian, Jing Meng, Shasha Qin, Xuan Tang, Fang Wang, Aihua Liang

**Affiliations:** ^1^ Key Laboratory of Beijing for Identification and Safety Evaluation of Chinese Medicine, Institute of Chinese Materia Medica, China Academy of Chinese Medical Sciences, Beijing, China; ^2^ National Engineering Laboratory for Quality Control Technology of Chinese Herbal Medicine, Institute of Chinese Materia Medica, China Academy of Chinese Medical Sciences, Beijing, China

**Keywords:** penilloic acid, penicillin, vascular leakage, arachidonic acid, non-allergic hypersensitivity reactions, RhoA/ROCK signaling pathway

## Abstract

Metabolites/impurities (MIs) of penicillin are normally considered to be the main substances inducing immediate hypersensitivity reactions in penicillin treatment. Our previous research found that penicillin can cause non-allergic hypersensitivity reactions (NAHRs) by directly triggering vascular hyperpermeability and exudative inflammation. However, the chief culprits and underlying mechanisms involved in penicillin-induced NAHRs have not yet been fully elucidated. In this study, we used a combination of approaches including a mouse non-allergic hypersensitivity reaction model, UPLC-MS/MS analyses of arachidonic acid metabolites (AAMs), immunoblotting technique, and molecular docking, etc to investigate the culprits involved in penicillin-induced hypersensitivity reactions. We found penilloic acid, one of the main MIs of penicillin, could trigger NAHRs via inducing increased vascular permeability, while the other MIs did no exhibit similar effect. Penilloic acid-induced reactions were not IgE-dependent. Significantly increased arachidonic acids and cascade metabolites in lungs, and activation of RhoA/ROCK signaling pathway in the ears and lungs of mice were noticed after once administration of penilloic acid. This study revealed that penilloic acid was the chief culprit involved in penicillin-induced immediate NAHRs in mice, which mainly associated with direct stimulation of vascular hyperpermeability and exudative inflammation. The activations of AAMs and RhoA/ROCK signaling pathway played important roles in these reactions.

## 1 Introduction

Penicillin is a widely used antibacterial drug with high antibacterial activity and low medical burden. However, penicillin allergy is self-reported by about 10% of the patients (Solensky, 2014; Vyles et al., 2020), which largely limits its application and increases the cost in clinic (Solensky, 2014; Castells et al., 2019). In fact, the incidence of true penicillin allergy is only estimated to be 1.1%–1.4% in adults and a little higher in children (Castells et al., 2019; Blanca-Lopez et al., 2021). The specific-IgE antibody can be detected in less than 10% of populations who claim to have a history of penicillin allergy, and most of them can tolerate using penicillin again (Solensky, 2012; Hjortlund et al., 2013; Macy, 2015). In previous study, we have put forward that penicillin can directly increase vascular permeability and induce non-allergic hypersensitivity reactions (NAHRs) (Han et al., 2016). This opinion somehow explains the low recurrence rate of penicillin allergy in clinical practice, and provides a new idea for the prevention and treatment of hypersensitivity reactions induced by penicillin.

NAHRs are non-immune-mediated hypersensitivity reactions, whose occurrence usually correlated with the dosage of the drugs. Typical symptoms of NAHRs, which include rash, itching, angioedema, bronchospasm, and anaphylaxis (Anterasian and Geng, 2018; Macy and Vyles, 2018; Banks et al., 2019), normally correlated with increased vascular leakage, changes in inflammation-related factors, and augmented exudation of tissue fluid. Mechanically, these reactions are triggered by direct degranulation of mast cells, activation of complement system, stimulation of inflammatory reaction-related enzymes and/or vascular permeability related pathways (Macy and Ensina, 2019).

RhoA/Rho kinase (ROCK) is an important signaling pathway regulates the formation of stress fibers and affects the permeability of blood vessels (Amano et al., 2010; Spindler et al., 2010; Tojkander et al., 2012). When RhoA forming an active GTP-bound state, it activates the downstream effector ROCK, triggers the activation of myosin light chain kinase (MLCK) and phosphorylation of myosin light chain phosphatase (MLCP), which results in rearrangement of F-actin cytoskeleton and disorder of endothelial function. Changes in the cytoskeleton of vascular endothelial cells can lead to increased endothelial permeability and cause dysfunction of vascular barrier. In our previous study, we found that RhoA/ROCK signaling pathway played an important role in penicillin-induced NAHRs (Han et al., 2016).

**FIGURE 1:**
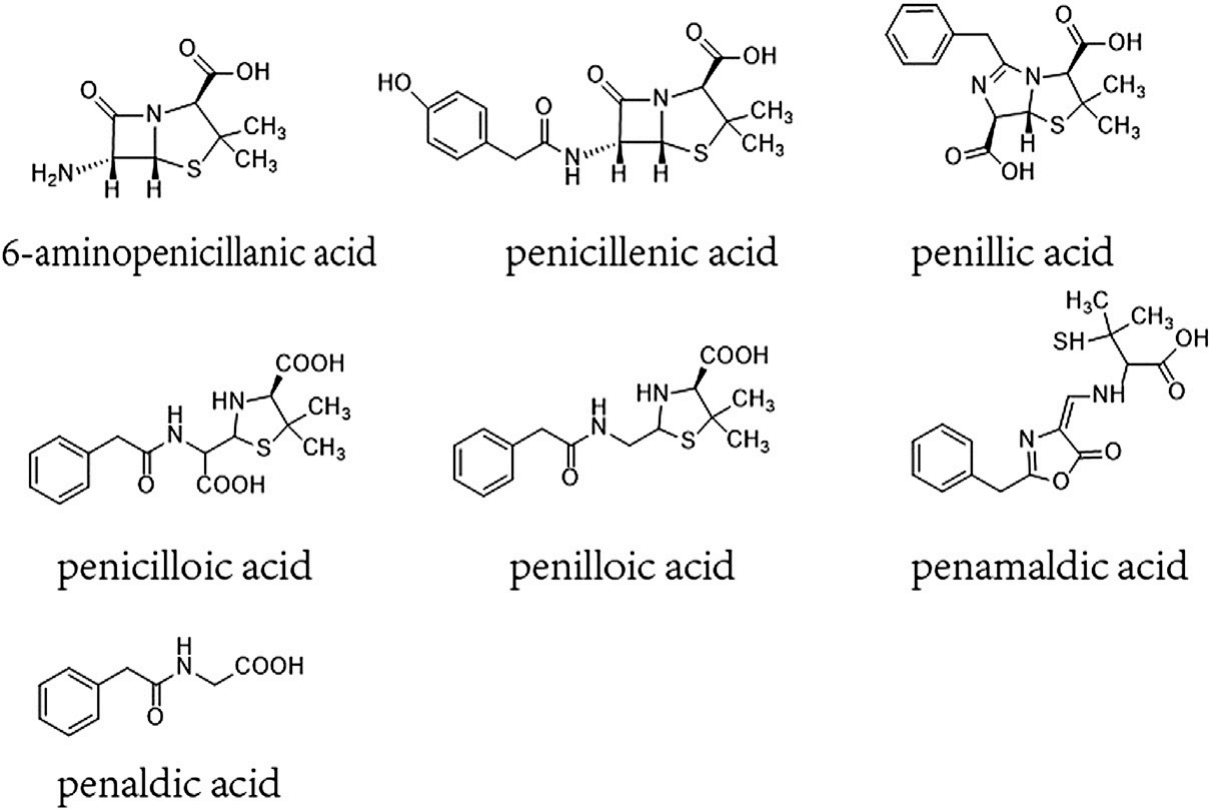
Chemical constructions of 6-aminopenicillanic acid, penicillenic acid, penillic acid, penicilloic acid, penilloic acid, penamaldic acid, penaldic acid.

β-lactam ring and its amide bond can break to form a series of metabolites such as penicillenic acid, penicilloic acid, penicillinic acid, penicillamine, penilloaldehyde, aminopenicillanic acid, penaldic acid, penamaldic acid, isopenicillic acid, penillic acid, penilloic acid, et al. (Ghebre-Sellassie et al., 1982; Li et al., 2008; Aldeek et al., 2016). These products can combine with amino or sulfhydryl groups of proteins and form hapten-biomolecule conjugates, which are normally considered to be the substances inducing allergic hypersensitivity reactions (AHRs) of penicillin (Baldo, 1999; Ariza et al., 2015). 6-aminopenicillanic acid, penicillenic acid, penillic acid, penicilloic acid, penilloic acid, penamaldic acid, and penaldic acid are also listed as main impurities contained in penicillin in both the European and Chinese pharmacopoeias. Their total content are limited to no more than 4% in penicillin product (Figure 1) (The European Pharmacopoeia Commission, 2016; National Commission of Chinese Pharmacopoeia, 2015). So far, penilloic acid, penicilloic acid and penicillamine are considered to be the main culprits leading to penicillin AHRs (Ho et al., 2011; Ariza et al., 2015). However, no investigation has been put forward to evaluate if these metabolites/impurities (MIs) can also induce NAHRs. In this study, we intent to search the MIs involved in immediate penicillin-induced NAHRs in mice and explore underlying mechanism.

## 2 Materials and methods

### 2.1 Reagents

Impurities of penicillin were commercially obtained at the purities ≥ 98% (HPLC analysis), including 6-aminopenicillanic acid (impurity A) was provided by Bailingwei (Beijing, China), penicillenic acid (impurity C) and penaldic acid (impurity I) were purchased from Saibaicao (Beijing, China), penillic acid (impurity D), penicilloic acid (impurity E) and penilloic acid (impurity F) were obtained from LGC (Germany), penamaldic acid (impurity H) was got from Toronto (Canada). Acetonitrile, methanol, isopropanol and formic acid were all purchased from Fisher Scientific (Waltham, MA, United States). The prostaglandins (6-keto-PGF_1α_, PGF_1α_, PGF_2α_, 15-keto-PGF_2_, PGJ_2_, 15-deoxy-12,14-PGJ_2_, 6-keto-PGE_1_, 13,14,dihydro-15-keto PGE_2_, PGD_2_, PGE_2_, TXB_2_, PGA_2_, PGI_2_, PGG_2_), leukotrienes (LXB_4_, LXA_4_, 20-hydroxy LTB_4_, LTB_4_, LTF_4_, LTD4, LTC4, LTE4), HETEs (5(S)-HETE, 12(S)-HETE, 15(S)-HETE, 15(S)-HpETE, 5(S)-HpETE, 12-HpETE), arachidonic acid (AA) and deuterated eicosanoids used as internal standards (PGE_2_-d_4_, LTE4-d_5_, 12-HETE-d_8_) were all purchased from Cayman Chemical Company (Ann Arbor, MI, United States). IL-6 ELISA kit was got from RD systems (United States). Ovalbumin (OVA) was obtained from Sigma-Aldrich (United States). Rabbit polyclonal antibodies against phospho-myosin light chain 2 (p-MLC2), myosin light chain 2 (MLC2), phosphorylated myosin phosphatase 1 (p-MYPT1), myosin phosphatase 1 (MYPT1), and monoclonal antibody against ras homolog family member A (RhoA) were all obtained from Cell Signaling Technology (Danvers, MA, United States). Rabbit polyclonal antibody against glyceraldehyde-3-phosphate dehydrogenase (GAPDH) was purchased from Santa Cruz Biotechnology (Santa Cruz, CA, United States). The activated RhoA pull-down assay kit was a product of Cytoskeleton (Denver, CO, United States). Goat anti-rabbit IgG antibody was got from ZSGB-BIO (Beijing, China). Nimesulide and Zileuton were provided by MedChemExpress (Monmouth Junction, NJ, United States), Fasudil hydrochloride was obtained from Chase Sun Pharmaceutical (Tianjin, China). Aluminum hydroxide gel was purchased from Thermo Fisher Scientific (Rockford, IL, United States).

### 2.2 Animals

Male ICR mice at 8–10 weeks of age were purchased from Vital River Laboratory Animal Technology Co., Ltd., Beijing. All mice were housed in a room with a constant temperature of 23°C–25°C and with a 12-h light/dark cycle. Mice were allowed free access to food and water. This study was approved by the Research Ethics Committee of the Institute of Chinese Materia Medica, China Academy of Chinese Medical Sciences, Beijing, China (Approval Number: 2019B035). All animal studies were carried out according to the recommendations of the ethical guidelines and regulations for the care and use of laboratory animals.

### 2.3 Assessment of vascular leakage in mice

#### 2.3.1 Vascular leakage test of penicillin from three different manufacturers

Twenty-four male mice were randomly divided into four groups (*n* = 6 per group) on a body weight-stratified basis. Normal saline or penicillin (500 kU/kg) from three different manufactures containing 0.4% EB were injected into the tail vein of mice parallelly. After 30 min of drug/EB treatment, vascular leakage was assessed by evaluating blue staining in the ears. A score from 1 to 6 was given by one researcher blinded to treatment of penicillin, where “1” denoted no visible blue area, and “2 to 6” represented visible blue areas of <12.5%, 12.5%–25%, 25%–50%, 50%–75%, and >75%, respectively. The ears of each mouse were preserved in 2 ml of formamide for EB extraction (Han et al., 2018; Pan et al., 2019b).

#### 2.3.2 Vascular leakage test of seven impurities of penicillin

Forty-eight male animals were randomly divided into eight groups (*n* = 6 per group). Impurities-treated mice received (iv) a single dose of 6-aminopenicillanic acid, penicillenic acid, penillic acid, penicilloic acid, penilloic acid, penamaldic acid, or penaldic acid at 12 mg/kg or normal saline with the same volume of 0.4% EB. Normal saline was treated as negative control. Vascular leakage was assessed by evaluating blue staining in the ears 30 min after drug/EB administration.

#### 2.3.3 Vascular leakage test for sensitization and unsensitization animals

Forty-eight male mice were randomly divided into eight groups (*n* = 6 per group). In the sensitization groups, animals were immunized by intraperitoneal injection (ip) of an equal volume of normal saline and aluminum hydroxide gel (NS/AHG), or penilloic acid (1.5, 3, 6 mg/kg)/AHG, on days 1, 3, 5, and 19, and challenged by injection (iv) of NS/EB or penilloic acid (3, 6, 12 mg/kg)/EB on day 33. In the unsensitization groups, naive mice received (iv) a single dose of NS/EB or penilloic acid (3, 6, 12 mg/kg)/EB at the same doses as those of challenge treatment in sensitized mice. Thirty minutes post drug/EB treatment, vascular leakage was assessed by evaluating blue staining in the ears.

### 2.4 Determination of IL-6 and NO in serum and inflammatory cells in lung

Twelve male mice were randomly divided into normal saline and penicilloic acid groups (*n* = 6 per group) and mice received (iv) a single dose of penicilloic acid (12 mg/kg) or normal saline. Thirty minutes post treatment, 1 ml blood and 1.5 ml lavage fluid were collected. The serum was tested for the contents of IL-6 and nitric oxide (NO) using ELISA and Griess assay, respectively. The lavage fluid was analyzed by cell count detection.

### 2.5 Histological examination

Ears and lungs of mice were fixed in 4% formalin and embedded in paraffin. Four-micrometer-thick serial sections were cut and stained with hematoxylin and eosin (HE). The sections were examined to evaluate inflammatory infiltration and structural damage.

### 2.6 Passive cutaneous anaphylaxis test

According to the method established in our previous research (Han et al., 2016; Han et al., 2018), serum from sensitized mice was obtained. Fifty microliters of serially diluted serum (1, 1/2, 1/4, and 1/8) was intradermally injected into the dorsal skin of depilated recipient mice. After 2 hours, the recipient mice were challenged by administration (iv) 0.4 ml of NS/EB, OVA (60 mg/kg)/EB, penilloic acid (12 mg/kg)/EB. Passive cutaneous anaphylaxis results were considered positive when the blue dorsal region had a diameter of 5 mm at 30 min after the challenge injection.

### 2.7 Immunoblot analysis

Samples were separated by SDS polyacrylamide gel electrophoresis (SDS-PAGE, 8%–12%). The separated proteins were subsequently transferred onto polyvinylidene fluoride (PVDF) membranes and blocked with skimmed milk at room temperature for 2 h, then blotted with indicated primary antibodies (1:500) overnight at 4°C. After washing, bound antibodies were detected with secondary goat anti-rabbit IgG antibody for 2 h at room temperature. The bands were probed using a chemiluminescence detection kit. We used Image J software to analyze blots Images.

### 2.8 Effect of nimesulide, zileuton and fasudil hydrochloride on penilloic acid-induced NAHRs

Thirty male mice were randomly divided into normal saline, nimesulide, zileuton or fasudil pretreatment groups, and penicilloic acid group (*n* = 6 per group). Animals from nimesulide, zileuton or fasudil pretreatment groups were given with 20 mg/kg nimesulide, 20 mg/kg zileuton, or 30 mg/kg fasudil hydrochloride (ip) in advance once daily for three consecutive days. On day 3, all mice received (iv) 12 mg/kg penilloic acid/EB at 30 min after the last dose of nimesulide, zileuton or fasudil. In the parallel group, mice were only treated (iv) with 12 mg/kg penilloic acid/EB. Thirty minutes after penilloic acid/EB injection, the scores of ears were evaluated.

### 2.9 Molecular docking

Auto Dock Vina was used for molecular docking and for calculating the binding score (Pan et al., 2019a). The GTP-RhoA analog complex was downloaded from the RCSB Protein Data Bank (PDB) using PDB ID:3TVD (Jobichen et al., 2012). Auto Dock Tool prepared this protein, and the active sites were predicted using the PyMOL Tool. The structures of compounds (6-aminopenicillanic acid, penicillenic acid, penillic acid, penicilloic acid, penilloic acid, N-phenylacetylglycine and 2-benzylpenicillenic) were drawn using Chem Draw Professional 18.0, and the structures were transferred to Chem3D. Then, the energy minimization of the structures MM2 force field was calculated, and the minimized ligands in PDB format were saved as PDBQT files using the Auto Dock Tool. The binding site of GTP-RhoA with a grid center was at x = 0.069, y = −36.86, z = 16.904. The number of points in each dimension was designed as X = 40, Y = 40, Z = 40. The interactions between GTP-RhoA and the compounds were visualized and analyzed using the PyMOL Visualization Tool.

### 2.10 Determination of impurities in penicillin by HPLC

HPLC analysis was performed on a Shimadzu LC-20A system (Kyoto, Japan) equipped with an SPD-20A photodiode array detector. Chromatographic separation was achieved on a Syncronis C18 column (5 μm, 250 mm × 4.6 mm) with a column temperature of 34°C using solvent A (phosphate buffer (PH 3.4)-methanol (72:14) and solvent B (acetonitrile). The injection volume was 10 μl. The mobile phase (delivered at 0.6 ml/min) consisted of solvent A and solvent B. Elution conditions were as follows: 0–42 min, B from 13.5% to 13.5%. UV absorbance was monitored at 225 nm for analysis.

### 2.11 Determination of AAMs in lungs by UPLC-MS/MS

Approximately 30 mg lung tissue was homogenized with 600 μl solvent (methanol:acetonitrile = 5:3) with 0.01 M butylated hydroxytoluene and 6 μl formic acid. The homogenized mixture was then centrifuged at 15,000 rpm at 4°C for 15 min. The supernatant was collected to dryness under nitrogen. Evaporated samples were reconstituted with 100 ul methanol (containing 5 μl of an internal standard (PGE2-d4, LTE4-d5, 12-HETE-d8) and subject to LC-MS analysis.

Quantitative determination of AAMs was performed with a UPLC-MS/MS system consisting of an ACQUITY UPLC I Class coupled to a Xevo-TQS detector (Waters, United States). Chromatographic separation was achieved on an ACQUITY UPLC®BEH C18 (100 mm × 2.1 mm, 1.7 μm), with column temperature of 40°C, auto-sampler temperature of 4°C, and the injection volume was 3 μl. The mobile phase (delivered at 0.45 ml/min) comprised 0.1% formic acid in water (A) and acetonitrile/isopropanol (90:10 v/v) (B). Elution conditions were as follows: 0–1 min, 25%–25% B; 1–4 min, 25%–33% B; 4–8 min, 33%–33% B; 8–14.5 min, 33%–48% B; 14.5–16.5 min, 48%–48% B; 16.5–19 min, 48%–52% B; 19–23 min, 52%–95% B; 23–23.5 min, 95%–95% B; 23.5–23.51 min, 95%–25%; 23.51–30 min, 25%–25% B. The total run time was 30 min.

Multiple reaction monitoring mode and negative electrospray ionization were applied for quantitative determination. The instrument parameters were as follows: source temperature, 150°C; capillary voltage, 2.5 kV; desolvation gas flow, 1,000 L/h; cone gas flow, 150 L/h; nebulizer gas, 7.0 Bar; desolvation temperature, 600°C. Details of the parent/daughter ions, cone voltages, and collision energies for 28 AAMs are listed in Supplementary Table 1.

### 2.12 Statistical analyses

All analyses were performed using SPSS 16.0 software. The results are described as the mean ± standard error of the mean (M ± SEM). Quantitative data among multiple groups were analyzed by one-way analysis of variance (ANOVA). The Rank-test analyzed the score of blue stain in the ears. *p* < 0.05 was considered to represent a statistically significant difference.

## 3 Results

### 3.1 Penilloic acid were the chief culprit involved in penicillin-induced NAHRs

**FIGURE 2:**
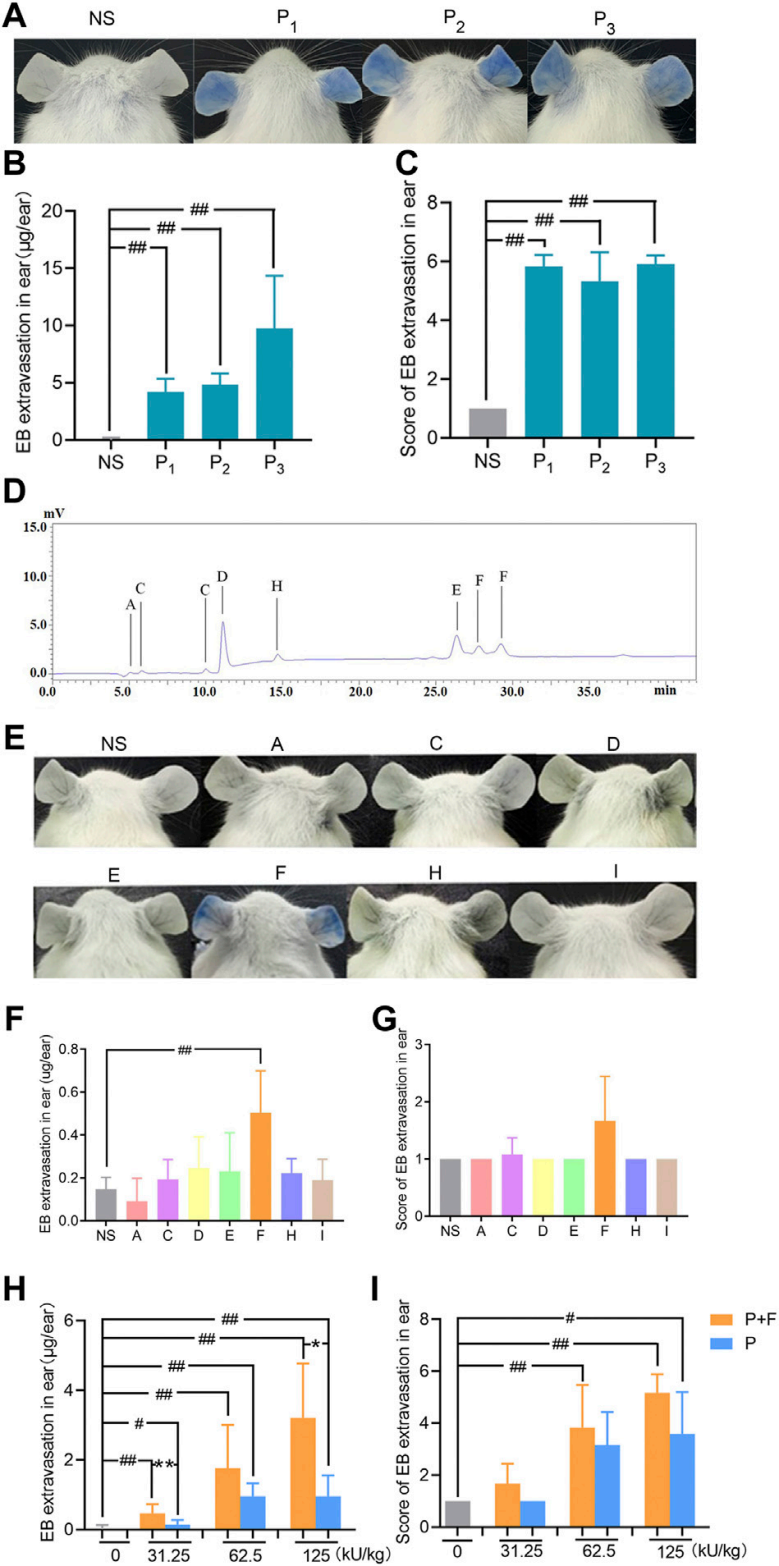
Penilloic acid was the chief culprit involved in penicillin-induced non-allergic hypersensitivity reactions (NAHRs). **(A–C)** Penicillin from different manufacturers induces same vascular leakage in mice. **(A)** Vascular leakage induced by penicillin (500 kU/kg) from three different manufactures, **(B)** EB extravasation and **(C)** score of EB staining in the ears (*n* = 6). NS represents normal saline; P_1_, P_2_, and P_3_ represent penicillin produced by three different manufacturers. **(D)** HPLC chromatogram of six Metabolites/impurities (MIs) in penicillin. **(E–G)** Among seven MIs, only penilloic acid can induce vascular hyperpermeability. **(E)** Vascular leakage in mice treated with seven MIs. A, C, D, E, F, H, I represents 6-aminopenicillanic acid, penicillenic acid, penillic acid, penicilloic acid, penilloic acid, penamaldic acid, penaldic acid, **(F)** EB extravasation and **(G)** score of EB staining in the ears (*n* = 6). **(H,I)** Penilloic acid aggravated vascular leakage induced by penicillin (*n* = 6). P + F: 31.25, 62.5 or 125 kU/kg penicillin with 6 mg/kg penilloic acid. P: single treatment of 31.25, 62.5 or 125 kU/kg penicillin. ^*#*^ *p* < 0.05 and ^*##*^ *p* < 0.01 compared with the NS group; *p < 0.05 and **p < 0.01 compared with the P + F-treated group.

In previous study, we have demonstrated that penicillin can directly induce systematic vascular hyperpermeability which was related to NAHRs in a mouse model within the equivalent clinical dose range (Han et al., 2016). To additionally exclude the influencing factors from pharmaceutical production, we firstly analyzed penicillin-induced vascular hyperpermeability of products from three different manufacturers by using the same model in this study. The result showed that all three penicillin products cause identical reactions of vascular hyperpermeability in mice, which could be easily observed by ear blue-staining (Figures 2A–C).

Metabolites/impurities (MIs) of penicillin have been considered to be the main substances involved in IgE-induced immediate hypersensitive reactions of penicillin (Weltzien and Padovan, 1998; Fernández et al., 2013). Both European and Chinese Pharmacopoeia have limited requirements for penicillin impurities (The European Pharmacopoeia Commission, 2016; National Commission of Chinese Pharmacopoeia, 2015). In this study, MIs in penicillin samples were analyzed by HPLC using seven commercially available MIs as references. Six types of MIs were determined in penicillin samples as shown in Figure 2D. We then tested if the MIs could directly cause vascular hyperpermeability. The dose used in the experiment were set according to the rang of clinical dosage of penicillin (human equivalent dose at 2.4 million units) and the maximum total content of impurities of penicillin limited in pharmacopoeias (4%). Among all tested MIs, apparent vascular leakage was only noticed in mice treated with penilloic acid. No visible change was observed in the other six MIs’ groups (Figures 2E–G). This result suggests that among seven MIs, only penilloic acid can directly induce vascular hyperpermeability.

In order to verify the contribution of penilloic acid on NAHRs of penicillin, a certain amount of penilloic acid was added to penicillin, and then we compared the difference in vascular hyperpermeability induced by penicillin and penicillin combined with penilloic acid. The result indicates that adding penilloic acid to penicillin could increase the vascular leakage caused by penicillin alone (Figures 2H,I), which also indicates penilloic acid contributes to penicillin-induced NAHRs.

### 3.2 Penilloic acid induced NAHRs was associated with direct inflammatory response

**FIGURE 3:**
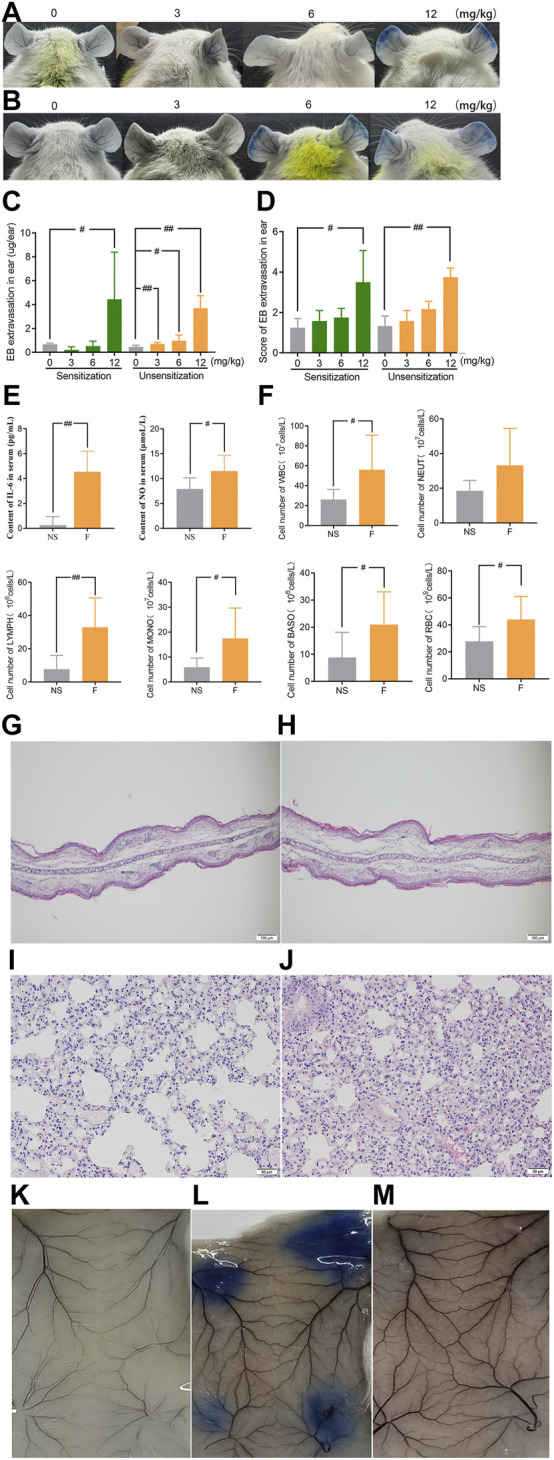
Penilloic acid-induced NAHRs was associated with directly inflammatory response without IgE-mediation. **(A–D)** Vascular leakage in the sensitized and unsensitized mice. **(A)** the sensitized mice, **(B)** the sensitized mice, **(C)** EB extravasation and **(D)** score of EB staining in the ears (*n* = 6). **(E)**The secretion of IL-6 and NO in serum (*n* = 6). **(F)** The lavage fluid of lung for cell count detection. ^*#*^ *p* < 0.05 and ^*##*^ *p* < 0.01 compared with the NS-treated group. **(G,H)** Microscopic examination of ears ×200. **(G)** NS, **(H)** penilloic acid (12 mg/kg). **(I,J)** Microscopic examination of lungs ×200. **(I)** NS, **(J)** penilloic acid (12 mg/kg). **(K–M)** Representative image of EB extravasation of mice dorsal inboard skin induced by penilloic acid (*n* = 3). **(K)** NS, **(L)** OVA, **(M)** penilloic acid (12 mg/kg).

In order to determine whether the vascular hyperpermeability caused by penilloic acid is immune-mediated response, we compared the degree of penilloic acid-induced vascular hyperpermeability in penilloic acid sensitized mice and unsensitized mice. Dose-dependent increased vascular hyperpermeability was observed both in sensitized mice and unsensitized mice. Prior sensitization did not enhance the extent of vascular leakage. Ear blue staining observed in sensitized and unsensitized mice treated (iv) with penilloic acid was extremely similar (Figures 3A–D). We further ruled out the role of penilloic acid-specific IgE in vascular leakage. As a positive control, OVA induced a 100% positive response, whereas no visible spot was appeared in penilloic acid-sensitized animals (Figures 3K–M). This result indicates that penilloic acid dose not elicit specific-IgE in mice in our experiment.

We further tested inflammatory factors involved in the IgE-independent reactions induced by penilloic acid. As shown in Figure 3E, IL-6 and NO in the serum of penilloic acid-treated mice were both significantly higher than those in NS group (*p* < 0.01, *p* < 0.05, respectively, compared to NS group). The total white blood cells, basophils, monocyte, and erythrocytes in lung lavage fluid were remarkably augmented under the stimulation of penilloic acid (*p* < 0.05 or *p* < 0.01, compared to NS group; Figure 3F). In histological examine, exudative inflammation was observed in the ears and lungs of mice in penilloic acid group. Edema with thickened auricle and sparse tissue structure were observed in ears (Figures 3G,H). Meanwhile, capillary congestion, inflammatory cell infiltration, alveolar septum thickening were occurred in lungs of penilloic acid-treated mice (Figures 3I,J). The results above reveal that penilloic acid may directly induce IgE-independent inflammatory responses and vascular hyperpermeability, which may trigger NAHRs.

### 3.3 Arachidonic acid metabolites were involved in penilloic acid-induced NAHRs

**FIGURE 4:**
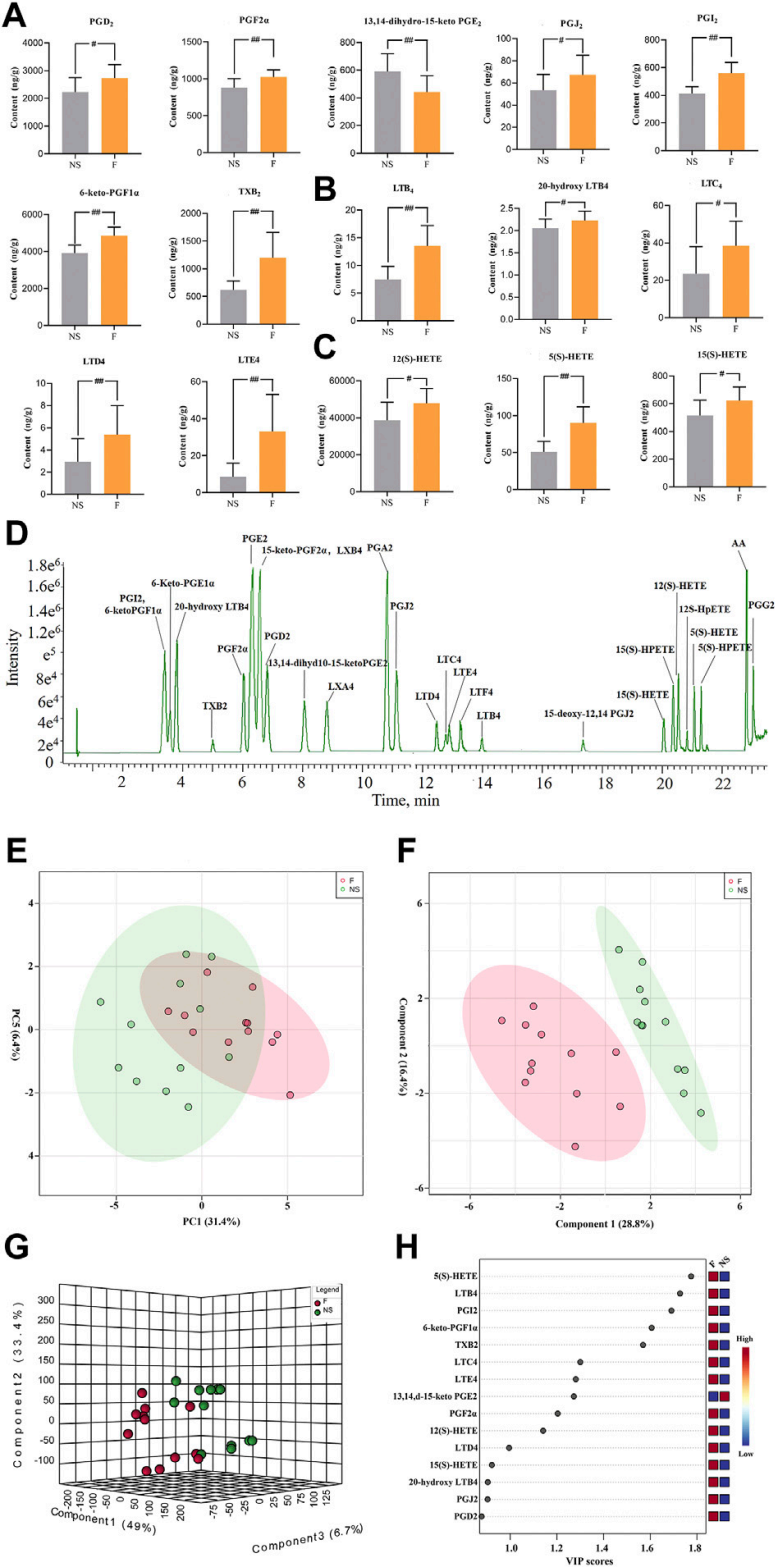
Determination of Arachidonic acid and cascade metabolites (AAMs) in lung. **(A–C)** Alterations in lung of the prostaglandins **(A)**, leukotrienes **(B)** and the HETEs**(C)** metabolites (VIP value > 0.8) after treated mice with penilloic acid (12 mg/kg) (*n* = 10). **(D)** LC-MS/MS chromatogram of 28 eicosanoids performed on a triple quadrupole employing dynamic MRM in negative mode. **(E)** PCA score plot for comprehensive metabonomics data for lung samples. **(F,G)** PLS-DA models of metabonomics data for lung samples. **(H)** The VIP score for major metabolites in lung.

Twenty-eight AAMs in lung tissues, including PGs, LTs, and HETEs were quantitatively measured in NS and penilloic acid groups using UPLC-MS/MS. Representative UPLC-MS/MS chromatograms of AAMs are shown in Figure 4D.

#### 3.3.1 Diversified analysis of pattern recognition of lung data

The statistical analysis module in Metaboanalyst 5.0 was used to calculate principal component analysis (PCA) and partial least squares discriminant analysis (PLS-DA). An initial PCA using the lung AAMs data revealed a partial segregation of penilloic acid and NS groups. It can be seen that there was a certain tendency for each group of samples to aggregate (Figure 4E). In order to obtain more ideal intergroup separation and enhance the identification of variables that contribute to the classification, a supervised PLS-DA analysis was further carried out. The model quality parameters were as follows: accuracy = 0.8333, R^2^ = 0.8357. These data indicate that this model is of modest quality and can provide accurate predictions. As shown in Figures 4F,G, the penilloic acid group was clearly separated from the NS group, indicating that AAMs levels in penilloic acid groups differed from control.

#### 3.3.2 Penilloic acid affected AAMs compositions

The variable importance in projection (VIP) values were used to identify the potential markers, and a VIP value above 0.8 was used as a cut of to select potential markers. Fifteen AAMs were obtained (Figure 4H). Quantitative analysis results of lung AAMs were showed in Figures 4A–C. Statistically significant increasdamounts of PGs (PGD_2_, PGJ_2_, PGF_2α_, 6-keto-PGF_1α_, PGI_2_, TXB_2_), LTs (LTE_4_, LTD_4_, LTC_4_, LTB_4_, 20-hydroxy-LTB_4_), HETEs (12(S)-HETE, 5(S)-HETE, 15(S)-HETE) and reduced levels of 13,14-dihydro-15-keto PGE_2_ were observed in penilloic acid group (*p* < 0.05 or *p* < 0.01, vs NS group). Notably, the penilloic acid group elevated the amounts of TXB_2_, LTB_4_, LTC_4_, LTD_4_, LTE_4_ by 93.7%, 80.4%, 63.8%, 84.8%, 290.8%, respectively.

The above results demonstrat that single administration of penilloic acid (iv) could activate AA metabolism. The increased the levels of the AA metabolites including PGs and LTs, especially LTs, are related with the inflammation exhibited in NAHRs.

### 3.4 Penilloic acid can active RhoA/ROCK signaling pathway

**FIGURE 5:**
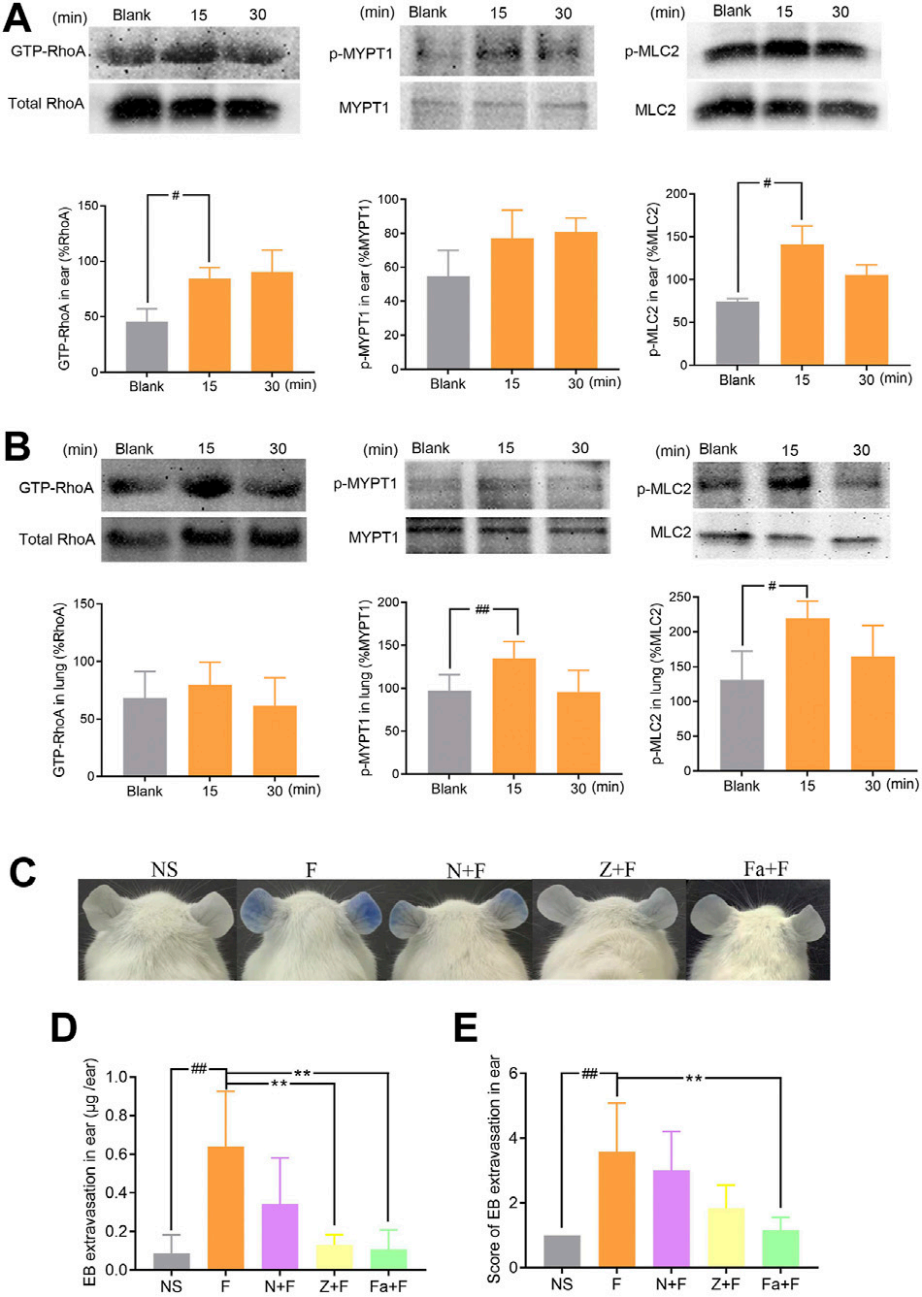
The effect of penilloic acid on RhoA/ROCK signaling pathway in mice. **(A)** Western analysis of GTP-RhoA/RhoA, p-MYPT1/MYPT1 and p-MLC2/MLC2 expression in ears of mice treated with 12 mg/kg penilloic acid for 15 min, 30 min, respectively (*n* = 3, per group). **(B)** Western analysis of GTP-RhoA/RhoA, p-MYPT1/MYPT1 and p-MLC2/MLC2 expression in lungs of mice treated with 12 mg/kg penilloic acid for 15 min, 30 min, respectively (*n* = 4, per group). **(C–E)** Nimesulide (COX-2 inhibitor), zileuton (5-LOX inhibitor) and fasudil (ROCK inhibitor) could reduce vascular leakage reaction induced by penilloic acid. **(D)** EB extravasation and **(E)** score of EB staining in the ears (*n* = 6). F: 12 mg/kg penilloic acid; N: 20 mg/kg nimesulide; Z: 20 mg/kg zileuton; Fa: 30 mg/kg fasudil. ^*#*^ *p* < 0.05 and ^*##*^ *p* < 0.01 compared with the NS group; *p < 0.05 compared with the penilloic acid group.

We next investigated the possible contribution of RhoA/ROCK signaling pathway in penilloic acid-induced NAHRs. Expressions of GTP-RhoA, p-MYPT1, and p-MLC2 in the ears and lungs were significantly up-regulated at 15 min after penilloic acid administration (Figures 5A,B). This result indicats that penilloic acid could activate RhoA/ROCK signaling pathway, which can cause endothelial barrier dysfunction and lead to vascular hyperpermeability.

We further explored the effect of fasudil (ROCK inhibitor), nimesulide (COX-2 inhibitor) and zileuton (5-LOX inhibitor) on penilloic acid-induced vascular leakage in mice. Pre-treatment with fasudil obviously inhibite the vascular leakage caused by penilloic acid, which also demonstrate that RhoA/ROCK signaling pathway contributed to penilloic acid-induced NAHRs. In addition, both nimesulide and zileuton could reduce penilloic acid-induced vascular leakage reaction (Figures 5C–E), which indicates that the inhibition of AA metabolism pathway may also patriciates in immediate penicillin NAHRs in mice.

**FIGURE 6:**
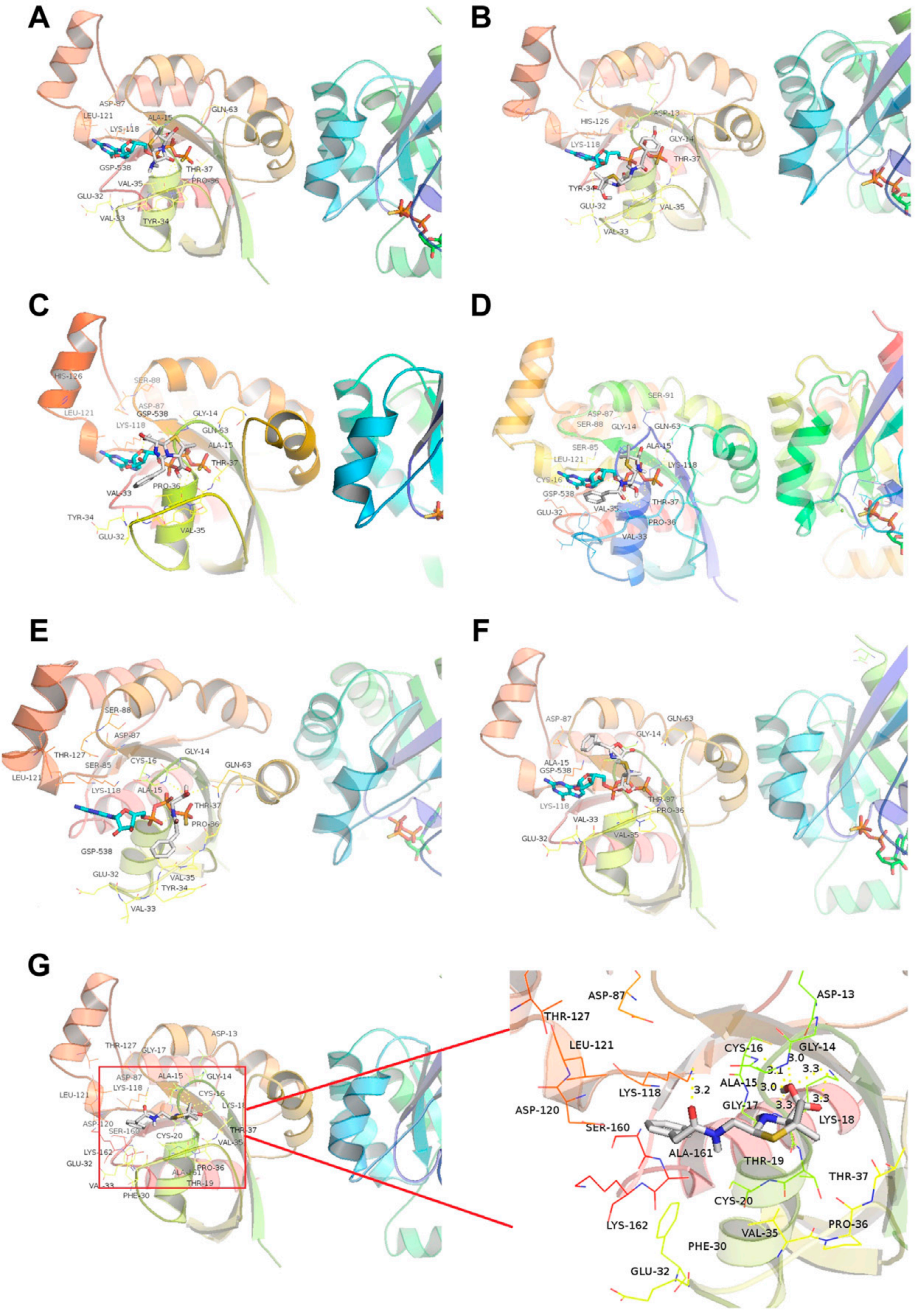
Docking of RhoA-GTPγS with the main metabolites/impurities of penicillin. **(A–F)** Substances that did not cause NAHRs: **(A)** 6-aminopenicillanic acid, **(B)** penicillenic acid, **(C)** penillic acid, **(D)** penicilloic acid, **(E)** penamaldic acid, **(F)** penaldic acid; **(G)** penilloic acid, which can induce NAHRs.

We then introduced relationship molecular modeling techniques to examine and visualize the interaction between penicillin impurities and RhoA/ROCK signaling pathway. 6-aminopenicillanic acid, penicillenic acid, penillic acid, penicilloic acid, penilloic acid, penamaldic acid, and penaldic acid were docked into the pivotal structural regions of RhoA-GTPγS. This was done using an induced fit procedure implemented in the Autodock Vina software. It is generally believed that the binding energy less than −5.0 kcal mol^−1^ indicates the ligand has better binding activity with the receptor, and less than −7.0 kcal mol^−1^ has strong binding activity (Hsin et al., 2013). The docking scores of 6-aminopenicillanic acid, penicillenic acid, penillic acid, penicilloic acid, penamaldic acid, penaldic acid and penilloic acid were −3.9, −5.5, −4.3, −4.7, −4.1, −4.2, and −7.0 kcal mol^−1^, respectively. Penilloic acid mainly interacted with amino acid residues such as LYS118, CYS16, ALA15, GLY14, ASP13, ALA161, LEU121, and SER160. When ligand interacted with protein, it mainly formed hydrogen bonds with LYS118, CYS16, GLY14, and LSY18, and the length of hydrogen bonds was 3.2, 3.1, 3.0, and 3.3, respectively (Figure 6). The formation of hydrogen bonds increases the ability of ligand to target protein. Penilloic acid is more likely to react with RhoA-GTPγS and form a stable interaction. This also verifies the ability of penilloic acid can activate the RhoA/ROCK signaling pathway.

## 4 Discussion

For a long time, people believe that the immediate-type hypersensitivity reaction caused by penicillin is mediated by IgE, named as type I allergic reaction. The penicilloyl group of penicillin is considered as major antigenic determinants, and a variety of other conjugates including penicillenate, penicilloic acid, penicillanyl, penamaldate, penaldate, d-penicillamine and penicoyl as minor determinants (Baldo, 1999). However, our previous studies proved that the first exposure of penicillin to mice can directly produce reactions characterized by the vascular hyperpermeability and exudative inflammation in organs including skin, lungs, etc. The pathological characteristics were likely to be NAHRs, because no IgE was proven to participate in the reactions (Han et al., 2016). Since NAHRs are not antigen related reaction, finding the chief culprits involved in penicillin-induced NAHRs are of high research significance. In the current study, single administration of penilloic acid could cause dose-dependent increased vascular permeability in mice, while no similar reaction was noticed in 6-aminopenicillanic acid, penicillenic acid, penaldic acid, penillic acid, penicilloic acid, and penamaldic acid treated animals. Penilloic acid also provoked obvious inflammatory exudation in ears and lungs of treated mice. Specific IgE could not be proven to participated in the reactions. These results indicate that penilloic acid can trigger NAHRs by directly eliciting vascular hyperpermeability and inflammation. Therefore, we consider that penilloic acid acts as one of the chief culprits causes immediate penicillin-induced NAHRs in mice.

The arachidonic acid (AA) pathway plays a key role in many inflammatory reactions, such as allergy, asthma, etc (Serrano-Mollar and Closa, 2005). Esterified AA on the inner surface of the cell membrane can be hydrolyzed to its free form by phospholipase A2 (PLA2), then further metabolized by cyclooxygenases (COXs) and lipoxygenases (LOXs) into a series of biologically active mediators, which mainly include PGs and LTs (Wang et al., 2021). AAMs including AA, various PGs (PGD_2_, PGJ_2_, PGF_2α_, 6-keto-PGF_1α_, PGI_2_, TXB_2_), LTs (LTE_4_, LTD_4_, LTC_4_, LTB_4_, 20-hydroxy-LTB_4_), HETEs (12(S)-HETE, 5(S)-HETE) are extremely high in lungs of mice treated with penilloic acid. It has been reported that various PGs and LTs play important roles in inflammatory reactions. PGE_2_ induces acute inflammation through mast cell activation via the EP3 receptor (Tsuge et al., 2019). PGD_2_ is related with mast cell degranulation (Matsuoka et al., 2000; Amorim et al., 2020), which has emerged as a central inflammatory lipid mediator associated with various allergy-associated disorders. PGD_2_ administration can directly upregulate vascular leakage in the skin and conjunctiva (Flower et al., 1976). Various LTs are potent endogenous chemotactic agent for neutrophils (Haeggström, 2018), which is a type of important inflammatory cells. The significant augmented in PGs and inflammatory cells both indicate that penilloic acid can trigger inflammation which are related with NAHRs.

The RhoA/ROCK signaling pathway is correlated with endothelial hyperpermeability and vascular leakage (Mikelis et al., 2015; Han et al., 2016). Our results demonstrate penilloic acid can up-regulate the expressions of p-MLC. Phosphorylation of MLC can induce actin cytoskeleton reorganization and lead to increasing vascular permeability (Etienne-Manneville and Hall, 2002; Wallez and Huber, 2008). As a result, blood cells effuse outside the blood vessels and cause interstitial edema and exudative inflammation, which, are both as observed in the ears and lungs of mice treated with penilloic acid. In addition, present data demonstrate that AAMs, such as AA, PGD_2_, PGF_2α_ provide extracellular signals that contribute to trigger the activation of RhoA/ROCK signaling pathway. Both COX-2 and 5-LOX inhibitor could reduce vascular leakage. Thus, the activation of RhoA/ROCK signaling pathway may be correlated with AAMs stimulation.

In summary, our results reveal that penilloic acid is the chief culprit involved in immediate penicillin-induced NAHRs in mice, which is mainly associated with vascular hyperpermeability and exudative inflammation. The activations of AAMs and RhoA/ROCK signaling pathway both play important roles in penilloic acid-induced NAHRs. It suggests that the content of penilloic acid in the penicillin product should be strictly controlled.

## Data Availability

The original contributions presented in the study are included in the article/Supplementary Material, further inquiries can be directed to the corresponding author.

## Abbreviations

AAMs, arachidonic acid metabolites; EB, evans blue; HE, hematoxylin and eosin; ip, intraperitoneal injections; iv, intravenous injection; MIs, metabolites/impurities; NAHRs, non-allergic hypersensitivity reactions; OVA, ovalbumin; PCA, principal component analysis; PLS-DA, partial least squares discriminant analysis.
