# Supplementary material for: Penilloic acid is the chief culprit involved in non-IgE mediated, immediate penicillin-induced hypersensitivity reactions in mice
Source: Front Pharmacol. 2022 Aug 22;13:874486. doi: 10.3389/fphar.2022.874486 (PMC9443931; doi:10.3389/fphar.2022.874486)
Supplement: Supplementary file 1 [file Table1.pdf]

Supplementary Table 1 List of the selected MRM parameters for each analytes

Analyte	Pathway	Q1 mass	Q3 mass	Cone	Collision
12(S)-HpETE	LOX	317.10	153.00	34	20
15(S)-HpETE	LOX	317.19	112.95	28	16
5(S)-HpETE	LOX	317.19	203.09	18	18
5(S)-HETE	LOX	319.20	114.90	18	16
12(S)-HETE	LOX	319.20	179.20	34	26
15(S)-HETE	LOX	319.20	219.04	28	14
LTB_4	LOX	335.20	195.10	6	14
LXA_4	LOX	351.19	114.89	40	14
20-hydroxy LTB_4	LOX	351.19	195.01	36	16
LXB_4	LOX	351.19	221.02	18	14
LTE_4	LOX	438.27	333.19	2	16
LTD_4	LOX	495.04	176.75	26	18
LTF_4	LOX	566.99	170.83	24	22
LTC_4	LOX	624.02	271.89	8	24
PGI_2	COX	350.87	350.87	2	4
PGA_2/PGJ_2	COX	333.18	271.12	34	12
PGE_2	COX	351.07	270.99	23	18
PGD2	COX	351.07	271.16	4	14
15-keto-$PGF_{2\alpha}$	COX	351.07	315.16	14	8
15-deoxy-12,14-PGJ2	COX	315.17	203.09	16	18
13,14,dihydro-15-keto PGE2	COX	351.19	315.15	14	18
$PGF_{2\alpha}$	COX	353.21	193.03	10	24
PGG_2	COX	367.00	367.00	2	4
6-keto-PGE_1	COX	367.12	331.16	2	12
6-keto-$PGF_{1\alpha}$	COX	368.82	368.82	2	4
TXB_2	COX	369.08	194.99	8	12
AA		303.30	295.10	27	12
12-HETE-d_8		327.27	184.05	32	14
LTE4-d_5		443.24	338.24	14	18
PGE2-d_4		355.28	275.19	2	16

measured
