# Supplementary material for: Penilloic acid is the chief culprit involved in non-IgE mediated, immediate penicillin-induced hypersensitivity reactions in mice
Source: Front Pharmacol. 2022 Aug 22;13:874486. doi: 10.3389/fphar.2022.874486 (PMC9443931; doi:10.3389/fphar.2022.874486)
Supplement: Supplementary file 2 [file Table1.docx]

Supplementary Table 1 List of the selected MRM parameters for each analytes measured

| Analyte | Pathway | Q1 mass | Q3 mass | Cone | Collision |
| --- | --- | --- | --- | --- | --- |
| 12(S)-HpETE | LOX | 317.10 | 153.00 | 34 | 20 |
| 15(S)-HpETE | LOX | 317.19 | 112.95 | 28 | 16 |
| 5(S)-HpETE | LOX | 317.19 | 203.09 | 18 | 18 |
| 5(S)-HETE | LOX | 319.20 | 114.90 | 18 | 16 |
| 12(S)-HETE | LOX | 319.20 | 179.20 | 34 | 26 |
| 15(S)-HETE | LOX | 319.20 | 219.04 | 28 | 14 |
| LTB_4_ | LOX | 335.20 | 195.10 | 6 | 14 |
| LXA_4_ | LOX | 351.19 | 114.89 | 40 | 14 |
| 20-hydroxy LTB_4_ | LOX | 351.19 | 195.01 | 36 | 16 |
| LXB_4_ | LOX | 351.19 | 221.02 | 18 | 14 |
| LTE_4_ | LOX | 438.27 | 333.19 | 2 | 16 |
| LTD_4_ | LOX | 495.04 | 176.75 | 26 | 18 |
| LTF_4_ | LOX | 566.99 | 170.83 | 24 | 22 |
| LTC_4_ | LOX | 624.02 | 271.89 | 8 | 24 |
| PGI_2_ | COX | 350.87 | 350.87 | 2 | 4 |
| PGA_2_/PGJ_2_ | COX | 333.18 | 271.12 | 34 | 12 |
| PGE_2_ | COX | 351.07 | 270.99 | 23 | 18 |
| PGD2 | COX | 351.07 | 271.16 | 4 | 14 |
| 15-keto-PGF_2α_ | COX | 351.07 | 315.16 | 14 | 8 |
| 15-deoxy-12,14-PGJ2 | COX | 315.17 | 203.09 | 16 | 18 |
| 13,14,dihydro-15-keto PGE2 | COX | 351.19 | 315.15 | 14 | 18 |
| PGF_2α_ | COX | 353.21 | 193.03 | 10 | 24 |
| PGG_2_ | COX | 367.00 | 367.00 | 2 | 4 |
| 6-keto-PGE_1_ | COX | 367.12 | 331.16 | 2 | 12 |
| 6-keto-PGF_1α_ | COX | 368.82 | 368.82 | 2 | 4 |
| TXB_2_ | COX | 369.08 | 194.99 | 8 | 12 |
| AA |  | 303.30 | 295.10 | 27 | 12 |
| 12-HETE-d_8_ |  | 327.27 | 184.05 | 32 | 14 |
| LTE4-d_5_ |  | 443.24 | 338.24 | 14 | 18 |
| PGE2-d_4_ |  | 355.28 | 275.19 | 2 | 16 |
