# Supplementary figures and images for: Penilloic acid is the chief culprit involved in non-IgE mediated, immediate penicillin-induced hypersensitivity reactions in mice
Source: Front Pharmacol. 2022 Aug 22;13:874486. doi: 10.3389/fphar.2022.874486 (PMC9443931; doi:10.3389/fphar.2022.874486)

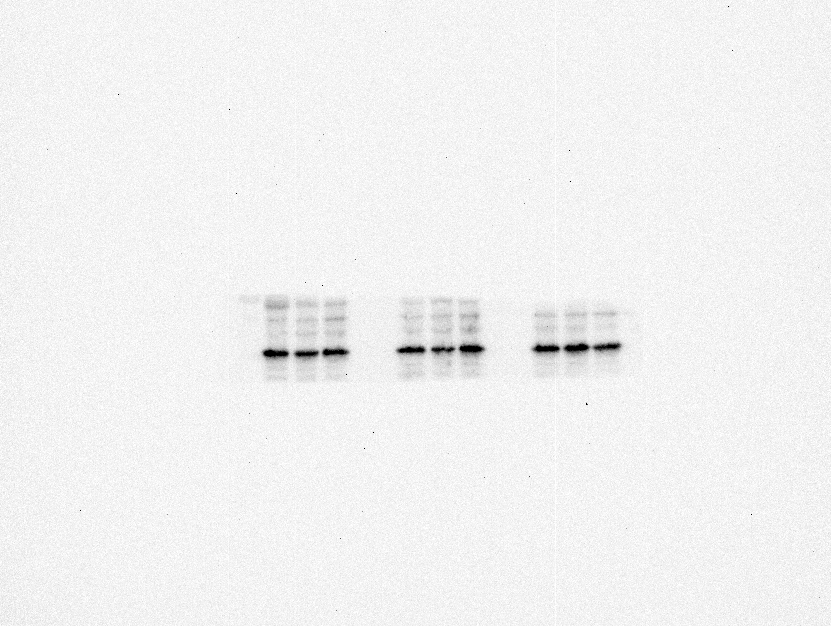

Supplement: Supplementary file 3 [file DataSheet1.ZIP › Original Images for Blots/WB-ear/GTP-RhoA and RhoA/GTP-RhoA/GAPDH.png]

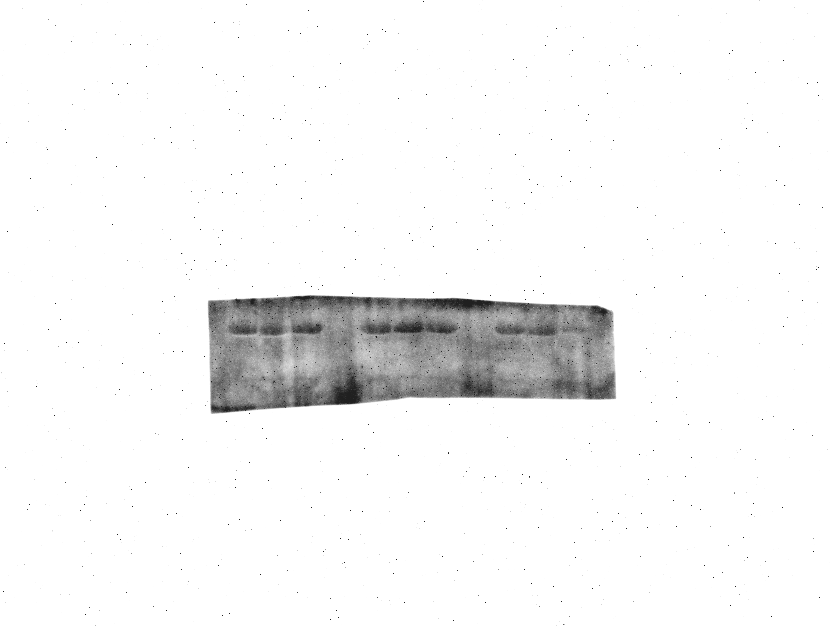

Supplement: Supplementary file 3 [file DataSheet1.ZIP › Original Images for Blots/WB-ear/GTP-RhoA and RhoA/GTP-RhoA/GTP-RhoA.png]

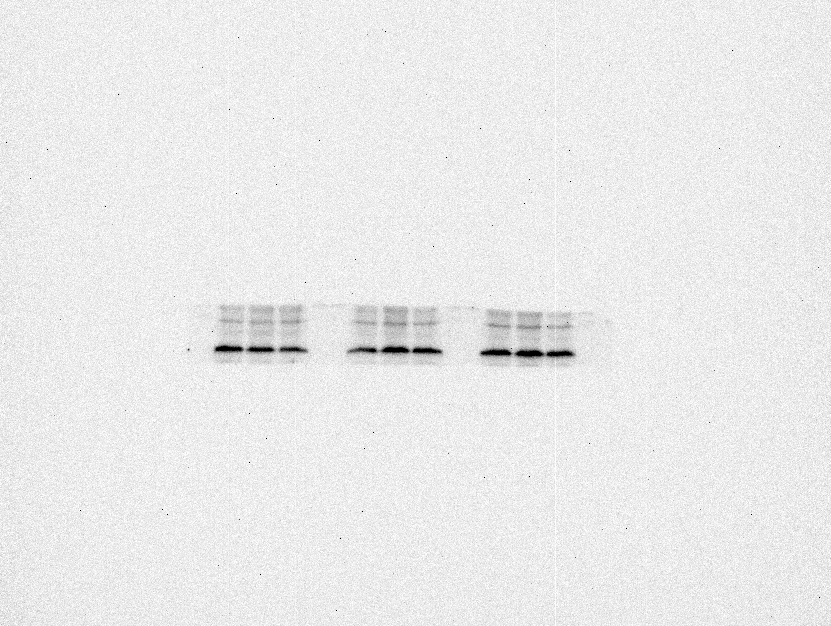

Supplement: Supplementary file 3 [file DataSheet1.ZIP › Original Images for Blots/WB-ear/GTP-RhoA and RhoA/RhoA/GAPDH.png]

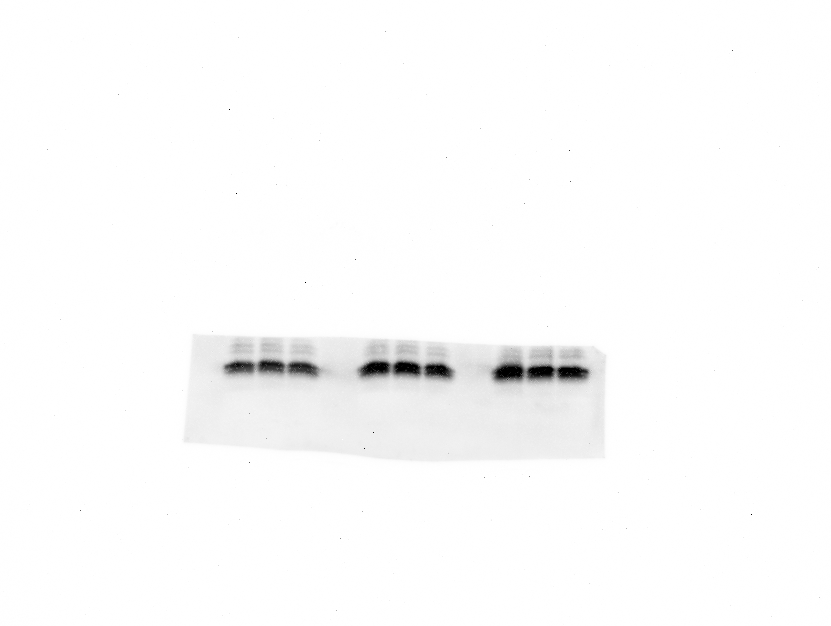

Supplement: Supplementary file 3 [file DataSheet1.ZIP › Original Images for Blots/WB-ear/GTP-RhoA and RhoA/RhoA/RhoA.png]

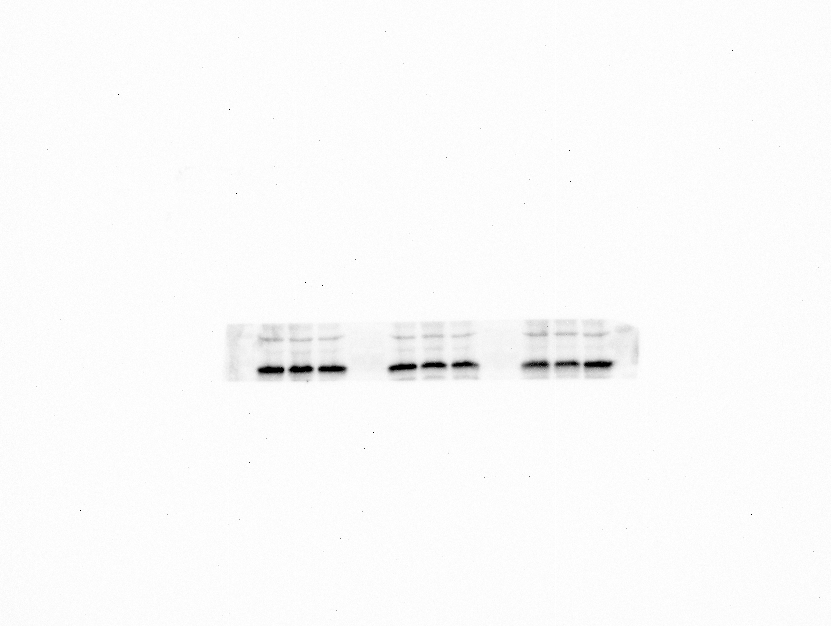

Supplement: Supplementary file 3 [file DataSheet1.ZIP › Original Images for Blots/WB-ear/P-MLC2 and MLC2/MLC2/GAPDH.png]

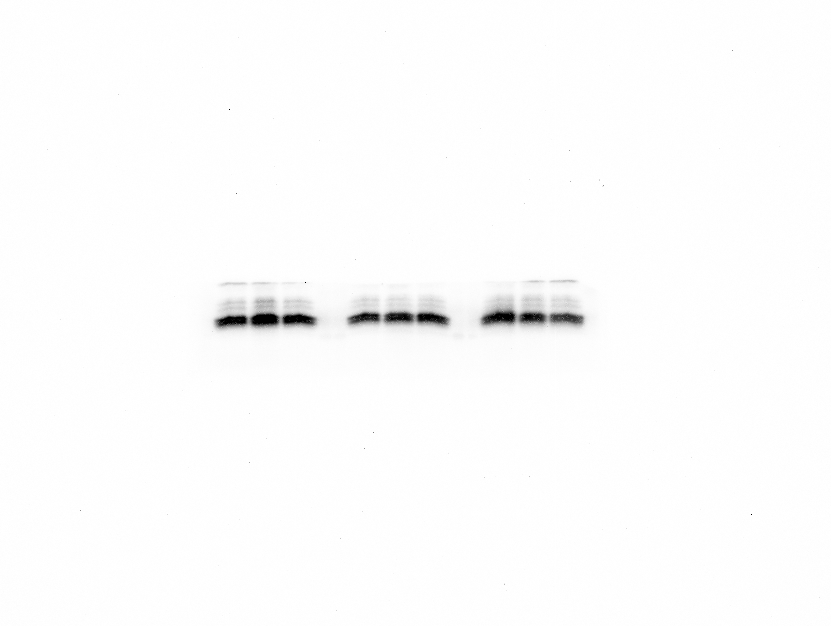

Supplement: Supplementary file 3 [file DataSheet1.ZIP › Original Images for Blots/WB-ear/P-MLC2 and MLC2/MLC2/MLC2.png]

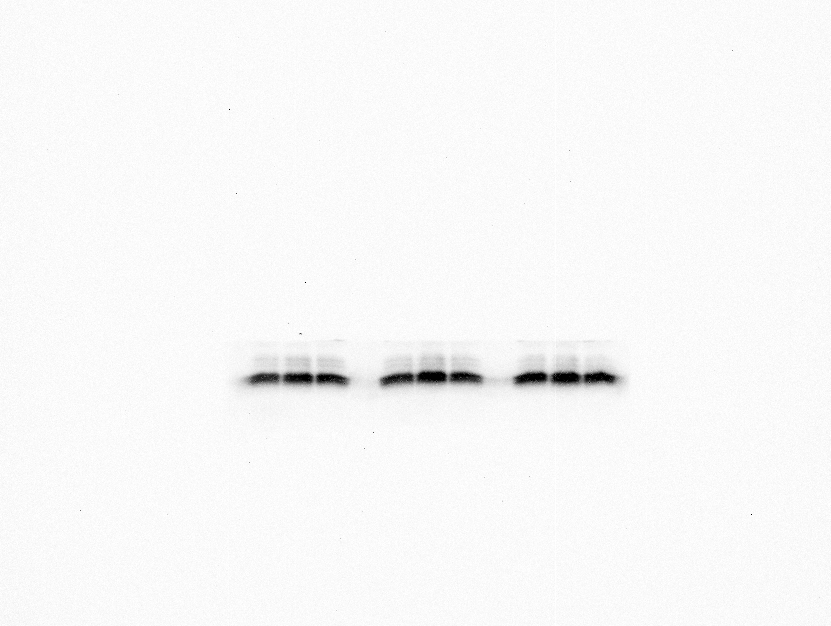

Supplement: Supplementary file 3 [file DataSheet1.ZIP › Original Images for Blots/WB-ear/P-MLC2 and MLC2/P-MLC2/p-MLC2.png]

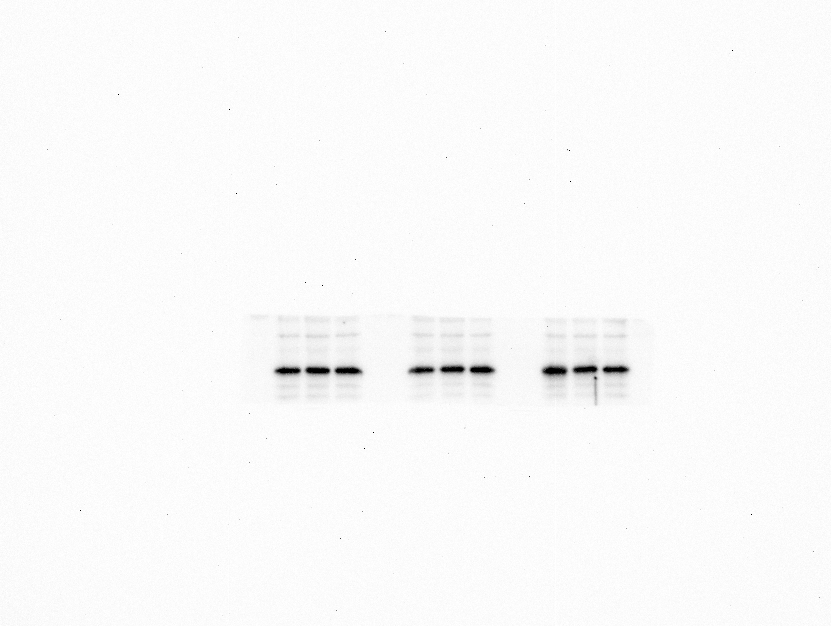

Supplement: Supplementary file 3 [file DataSheet1.ZIP › Original Images for Blots/WB-ear/P-MYPT1 and MYPT1/MYPT1/GAPDH.png]

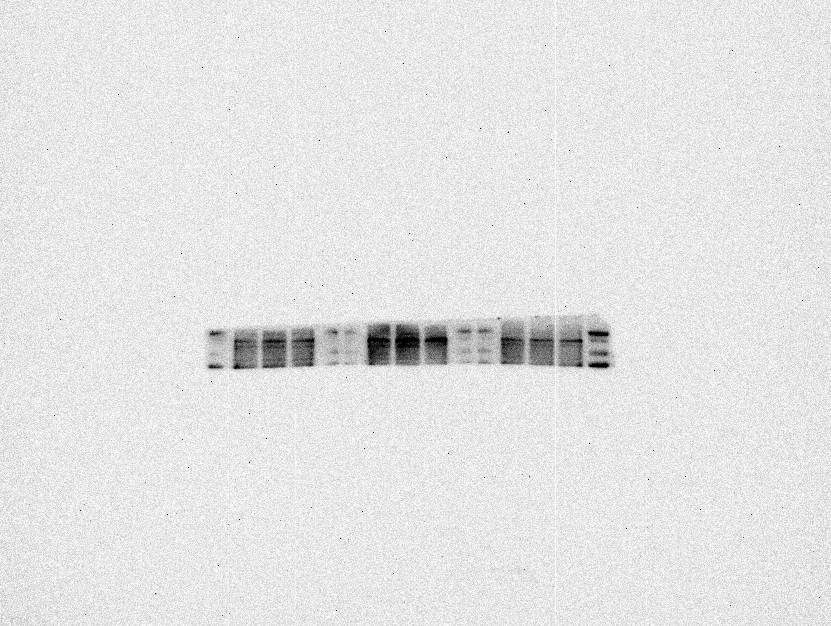

Supplement: Supplementary file 3 [file DataSheet1.ZIP › Original Images for Blots/WB-ear/P-MYPT1 and MYPT1/MYPT1/MYPT1.png]

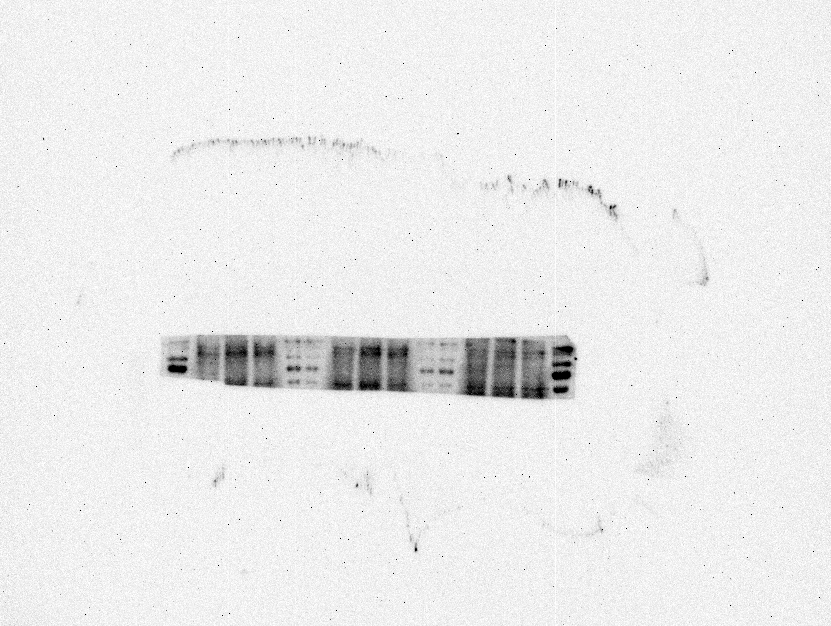

Supplement: Supplementary file 3 [file DataSheet1.ZIP › Original Images for Blots/WB-ear/P-MYPT1 and MYPT1/P-MYPT1/P-MYPT1.png]

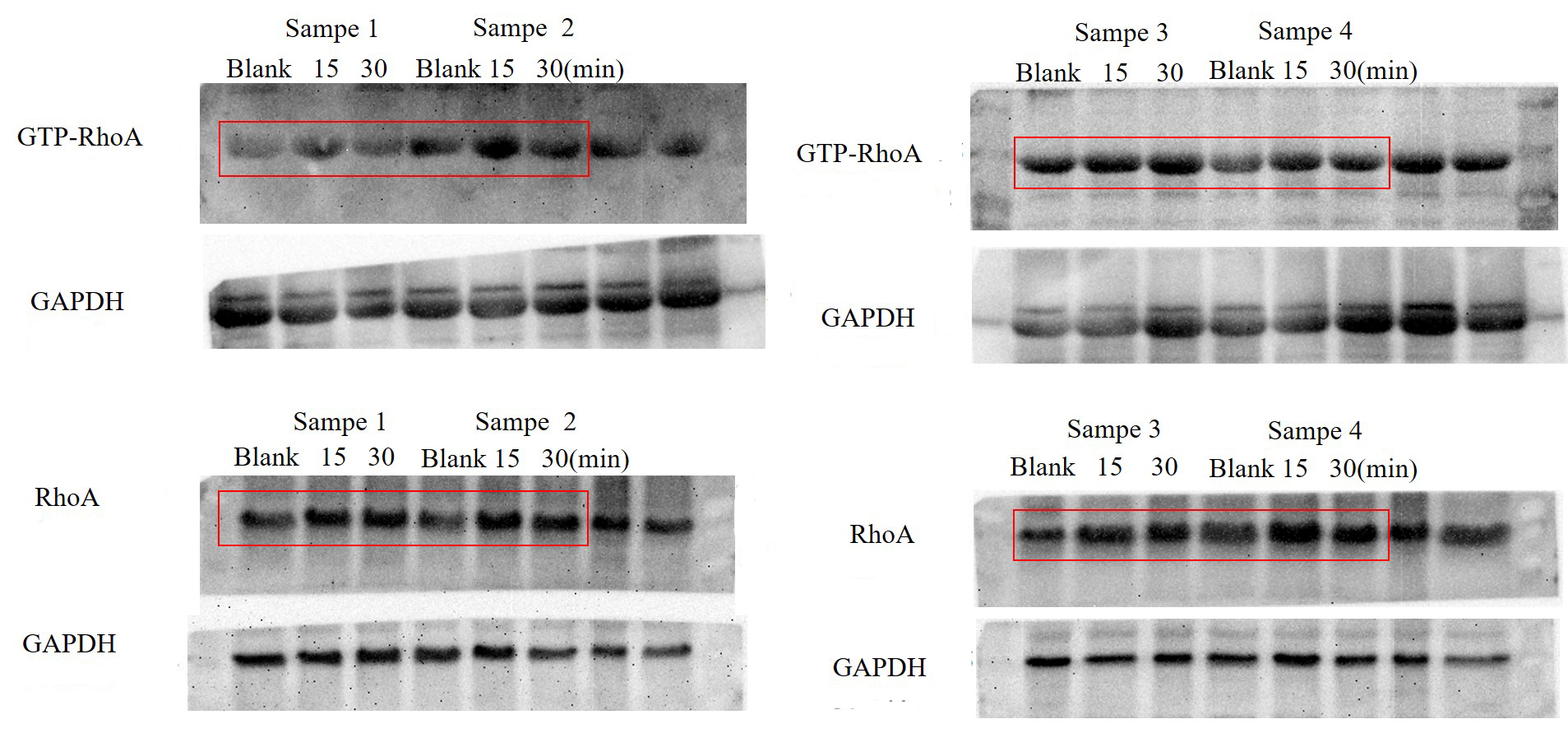

Supplement: Supplementary file 3 [file DataSheet1.ZIP › Original Images for Blots/WB-lung/Figure-GTP-RhoA and RhoA.jpg]

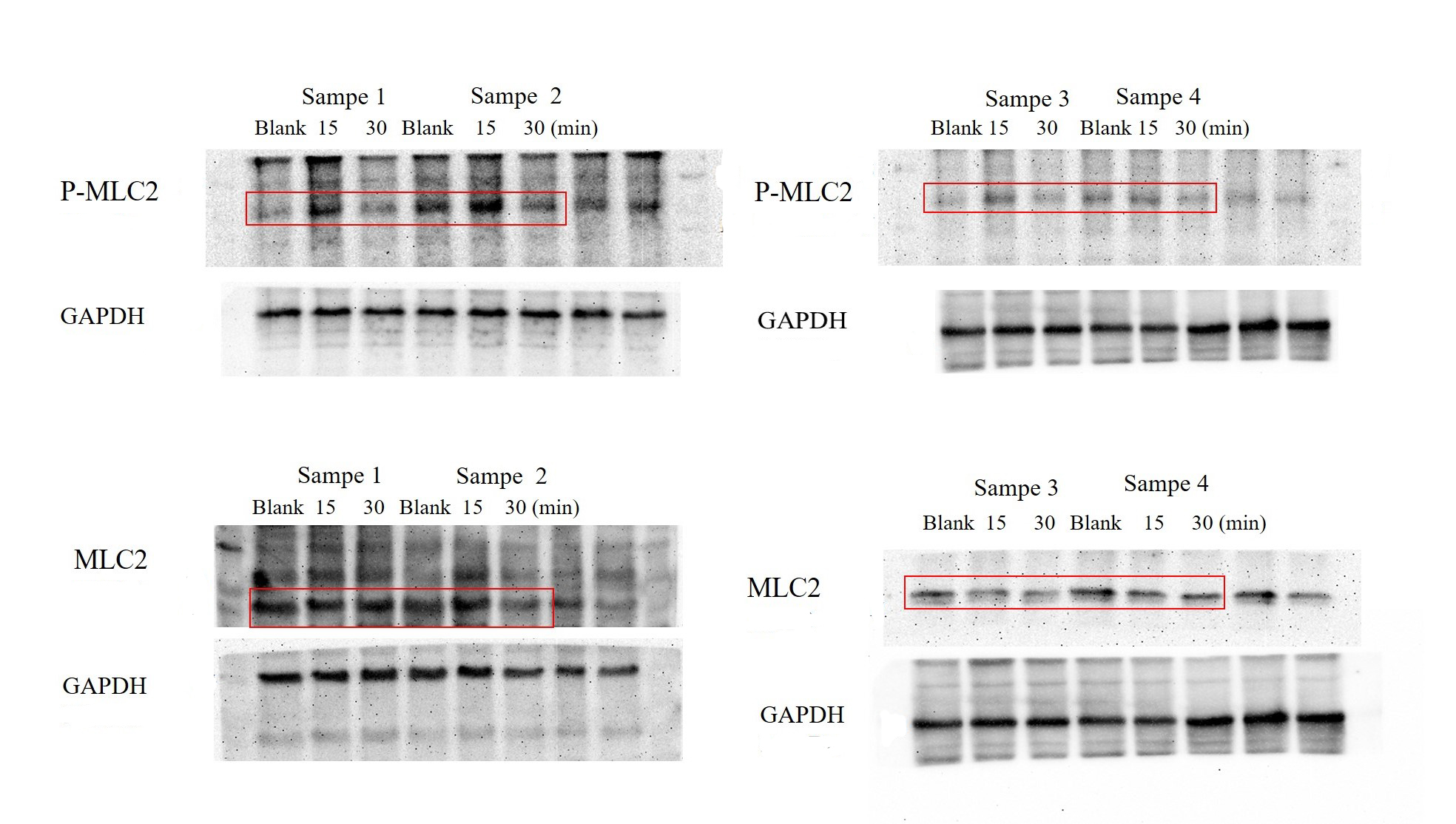

Supplement: Supplementary file 3 [file DataSheet1.ZIP › Original Images for Blots/WB-lung/Figure-P-MLC2 and MLC2.jpg]

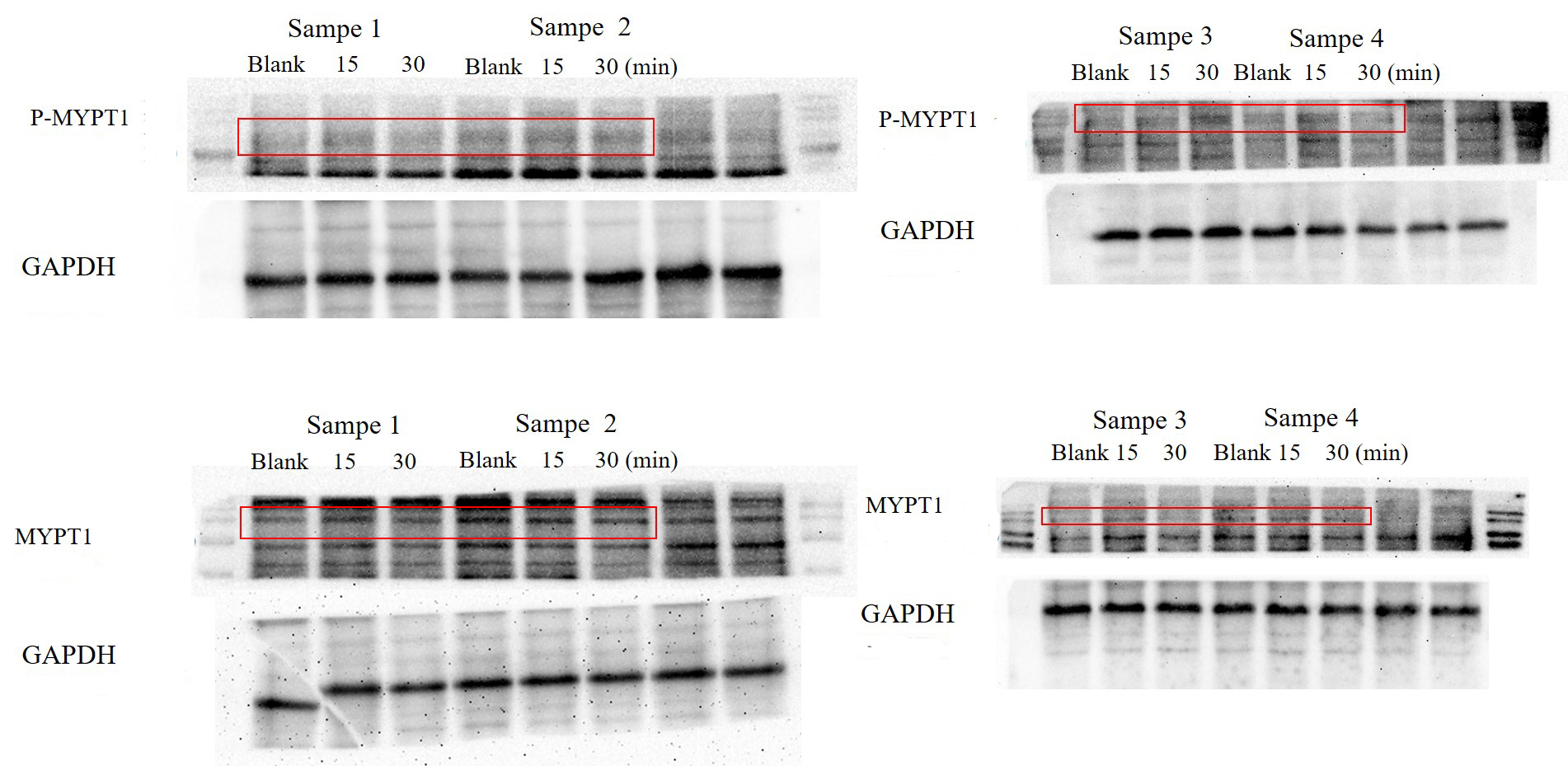

Supplement: Supplementary file 3 [file DataSheet1.ZIP › Original Images for Blots/WB-lung/Figure-P-MYPT1 and MYPT1.jpg]

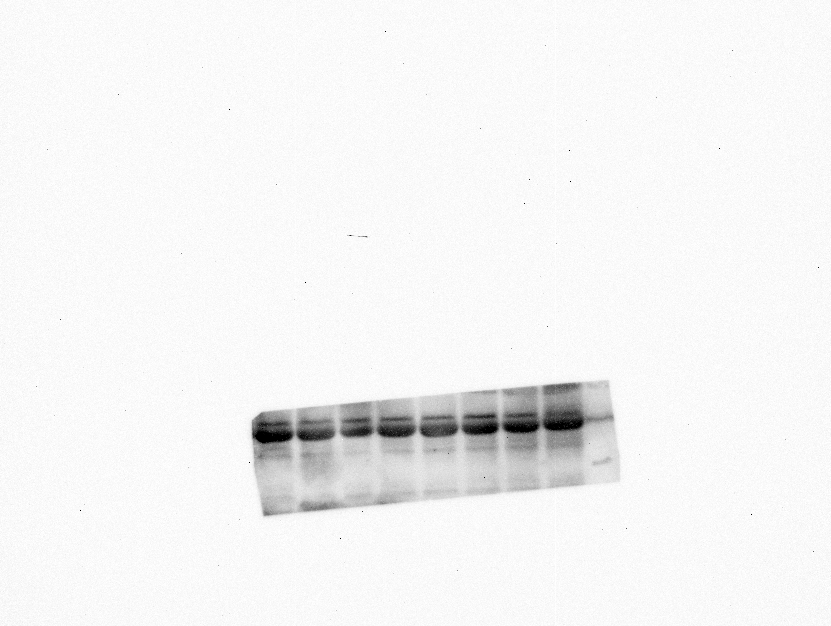

Supplement: Supplementary file 3 [file DataSheet1.ZIP › Original Images for Blots/WB-lung/GTP-RhoA and RhoA/GTP-RhoA/GTP-GAPDH-1.png]

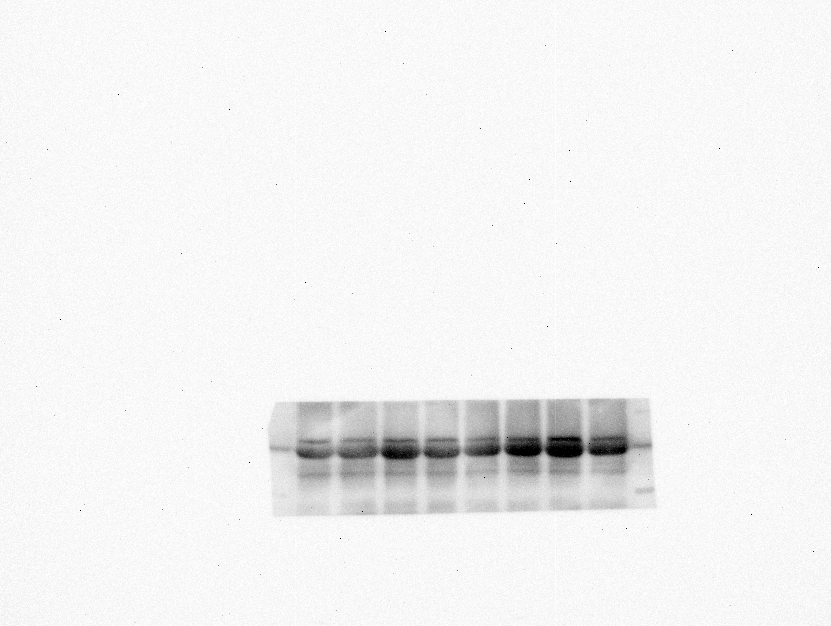

Supplement: Supplementary file 3 [file DataSheet1.ZIP › Original Images for Blots/WB-lung/GTP-RhoA and RhoA/GTP-RhoA/GTP-GAPDH-2.png]

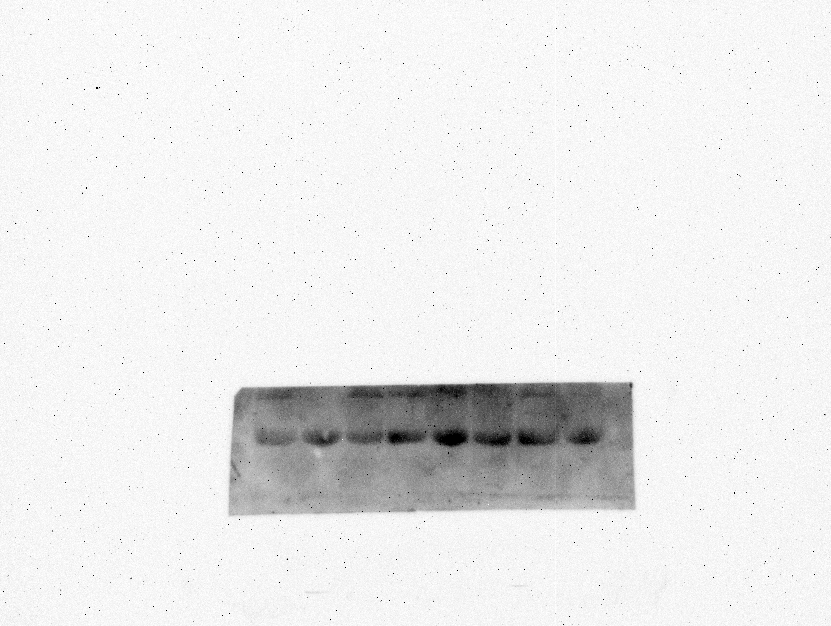

Supplement: Supplementary file 3 [file DataSheet1.ZIP › Original Images for Blots/WB-lung/GTP-RhoA and RhoA/GTP-RhoA/GTP-RhoA-1.png]

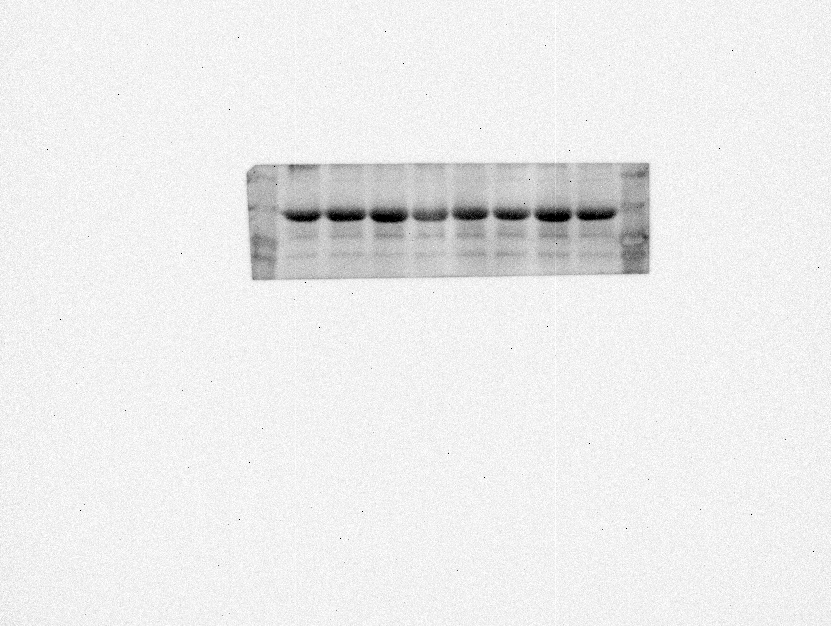

Supplement: Supplementary file 3 [file DataSheet1.ZIP › Original Images for Blots/WB-lung/GTP-RhoA and RhoA/GTP-RhoA/GTP-RhoA-2.png]

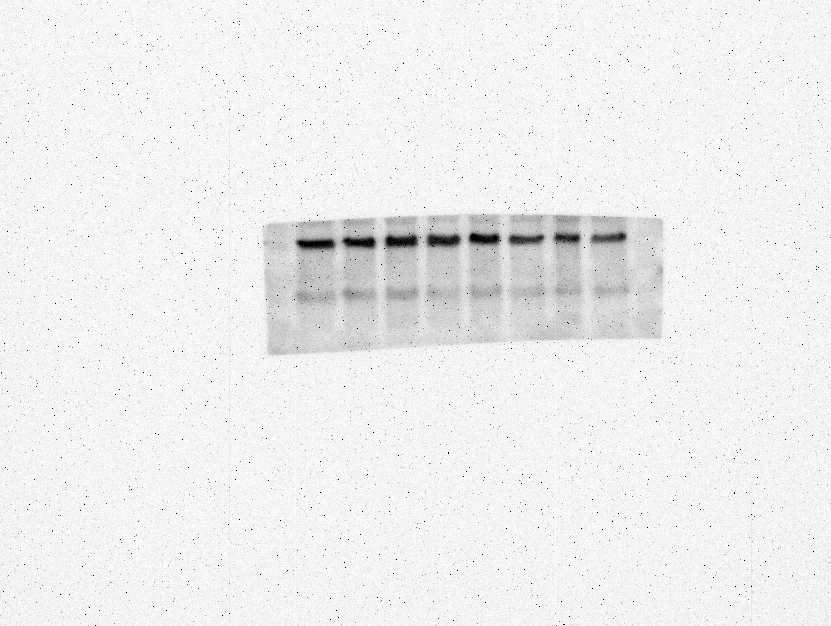

Supplement: Supplementary file 3 [file DataSheet1.ZIP › Original Images for Blots/WB-lung/GTP-RhoA and RhoA/RhoA/GAPDH-1.png]

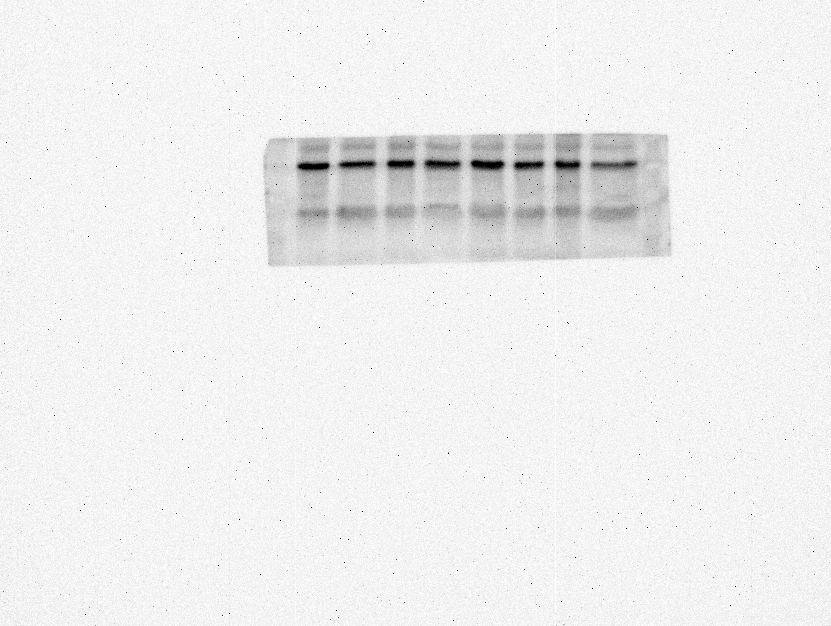

Supplement: Supplementary file 3 [file DataSheet1.ZIP › Original Images for Blots/WB-lung/GTP-RhoA and RhoA/RhoA/GAPDH-2.png]

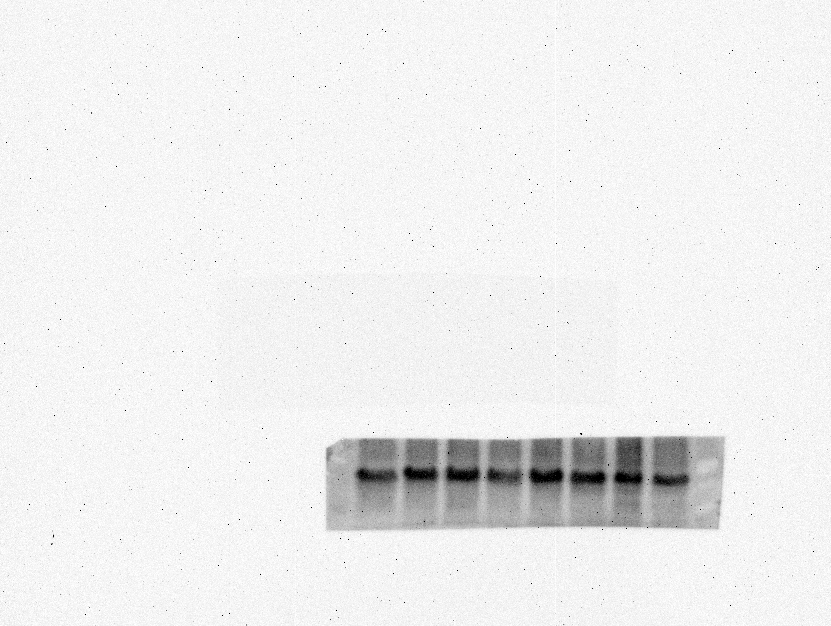

Supplement: Supplementary file 3 [file DataSheet1.ZIP › Original Images for Blots/WB-lung/GTP-RhoA and RhoA/RhoA/RhoA-1.png]

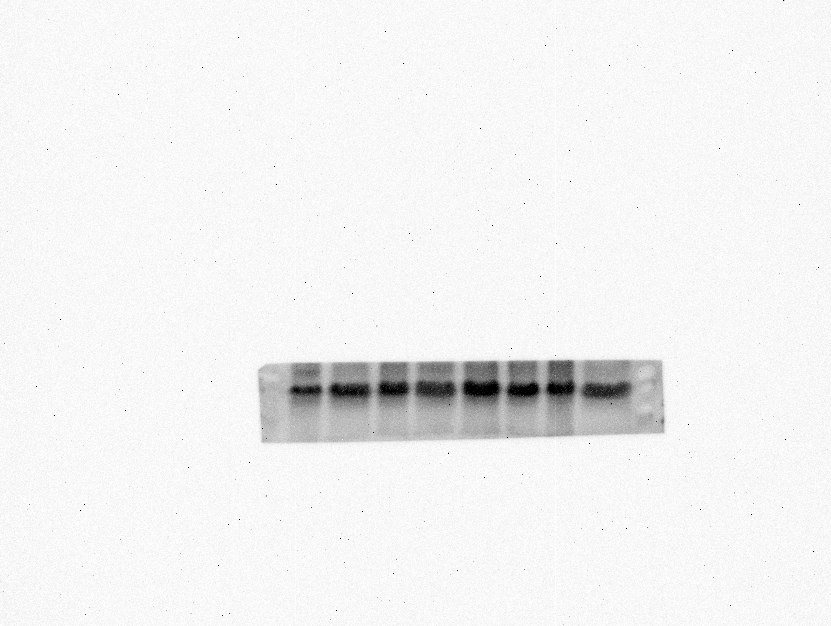

Supplement: Supplementary file 3 [file DataSheet1.ZIP › Original Images for Blots/WB-lung/GTP-RhoA and RhoA/RhoA/RhoA-2.png]

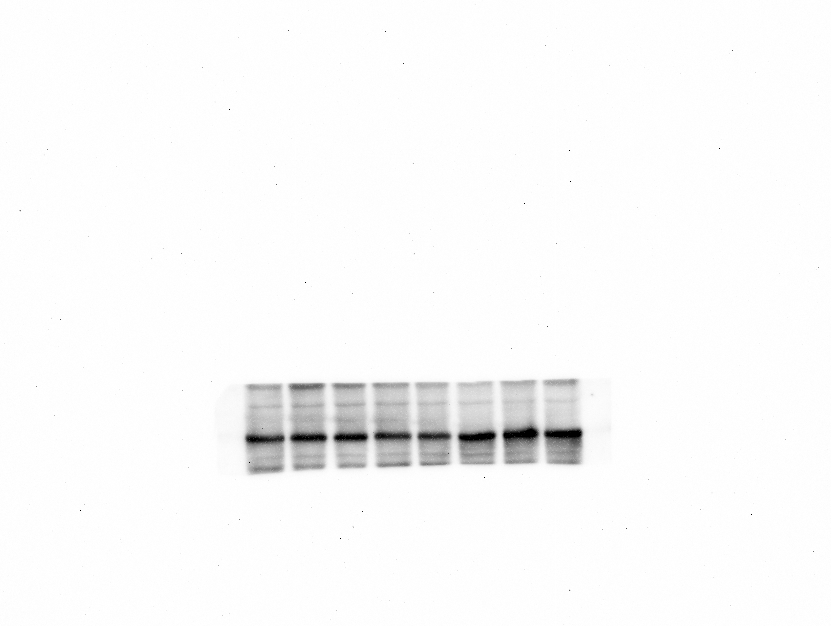

Supplement: Supplementary file 3 [file DataSheet1.ZIP › Original Images for Blots/WB-lung/P-MLC2 and MLC2/MLC2/GAPDH-2.png]

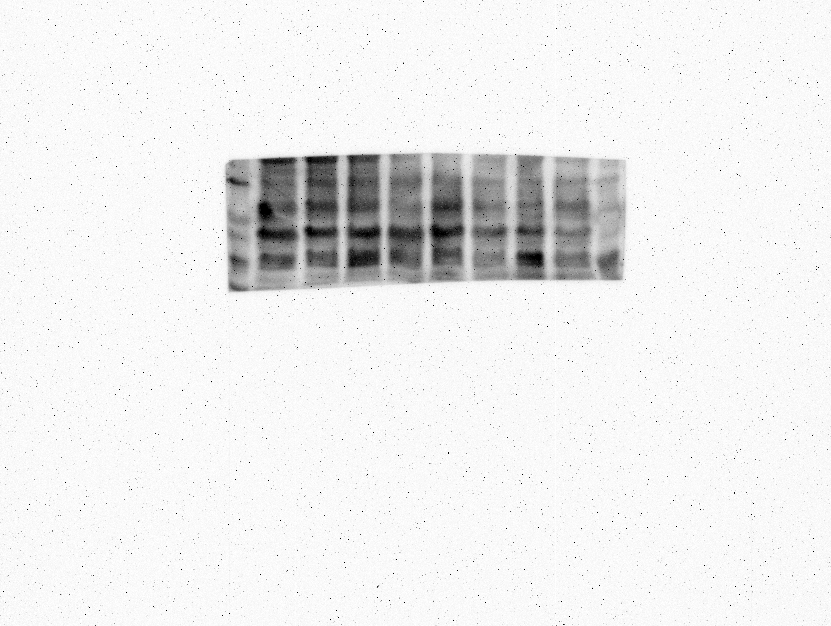

Supplement: Supplementary file 3 [file DataSheet1.ZIP › Original Images for Blots/WB-lung/P-MLC2 and MLC2/MLC2/MLC2-1.png]

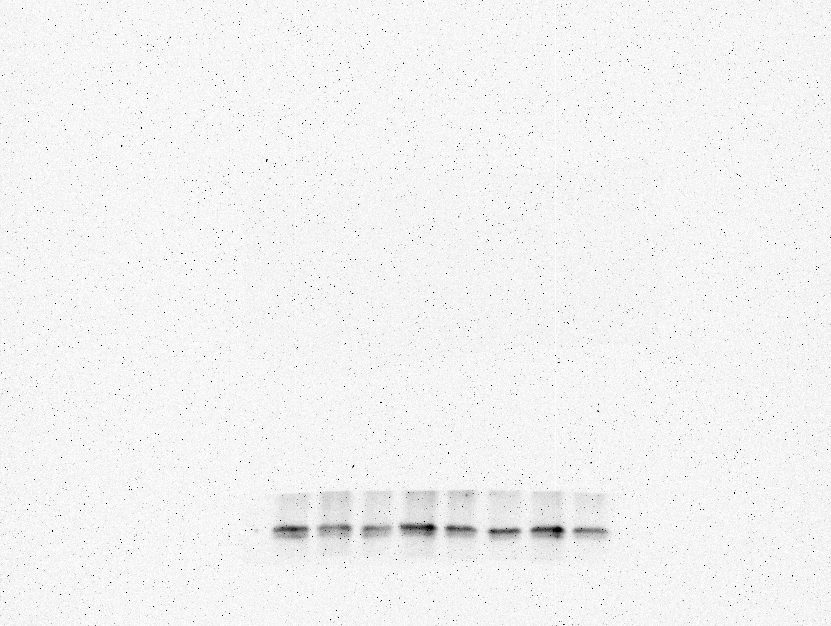

Supplement: Supplementary file 3 [file DataSheet1.ZIP › Original Images for Blots/WB-lung/P-MLC2 and MLC2/MLC2/MLC2-2.png]

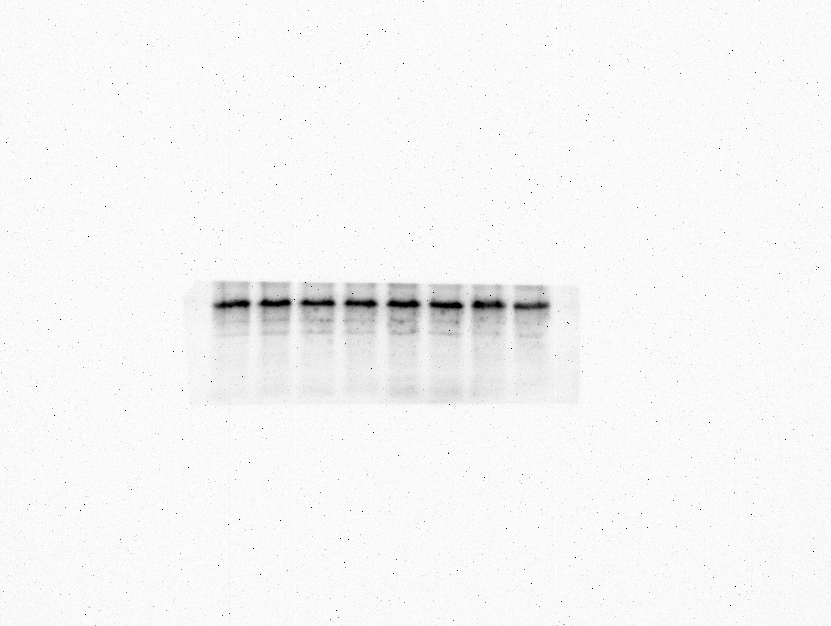

Supplement: Supplementary file 3 [file DataSheet1.ZIP › Original Images for Blots/WB-lung/P-MLC2 and MLC2/P-MLC2/GAPDH-1.png]

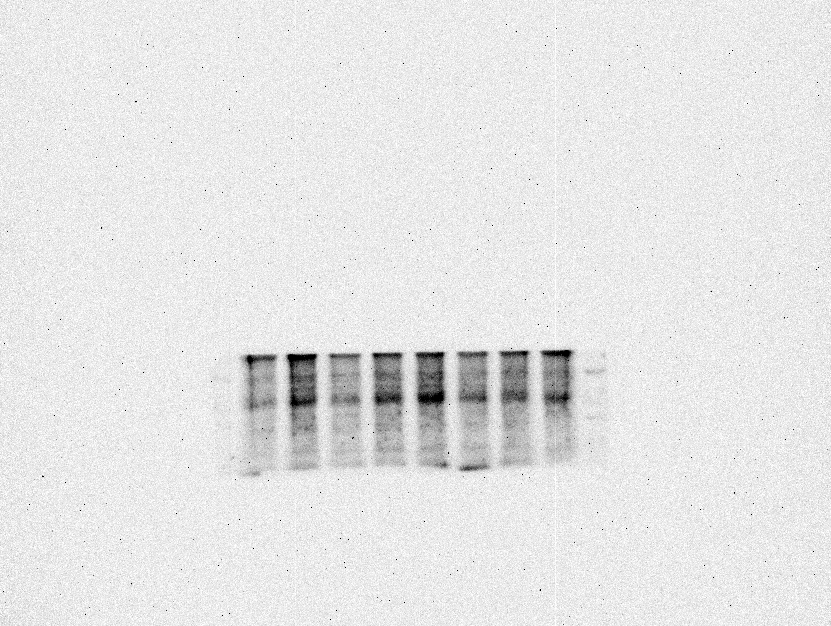

Supplement: Supplementary file 3 [file DataSheet1.ZIP › Original Images for Blots/WB-lung/P-MLC2 and MLC2/P-MLC2/P-MLC2-1.png]

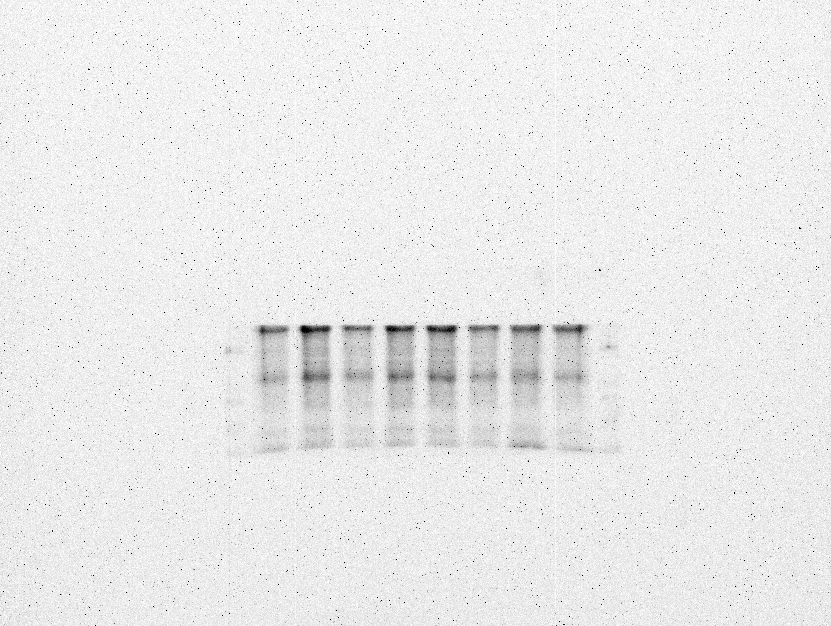

Supplement: Supplementary file 3 [file DataSheet1.ZIP › Original Images for Blots/WB-lung/P-MLC2 and MLC2/P-MLC2/P-MLC2-2.png]

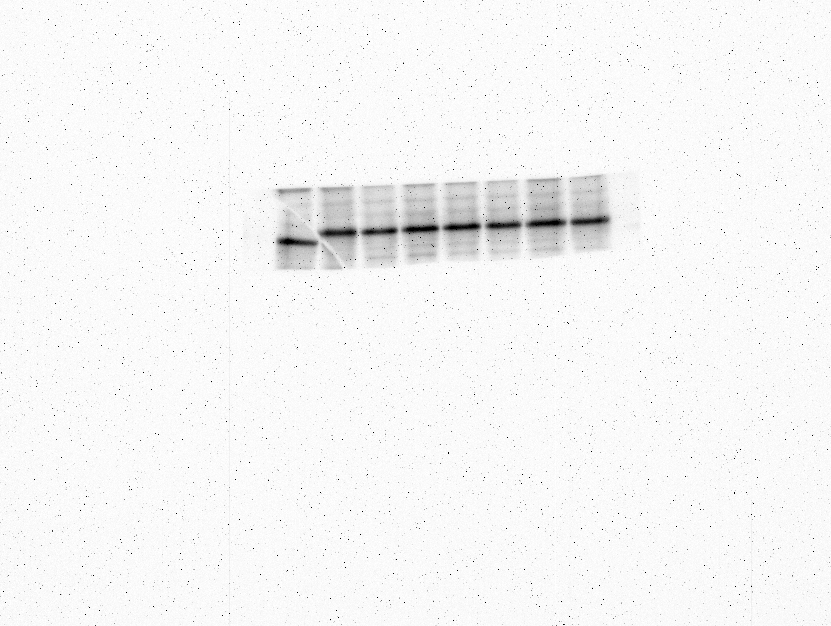

Supplement: Supplementary file 3 [file DataSheet1.ZIP › Original Images for Blots/WB-lung/P-MYPT1 and MYPT1/MYPT1/GAPDH-1.png]

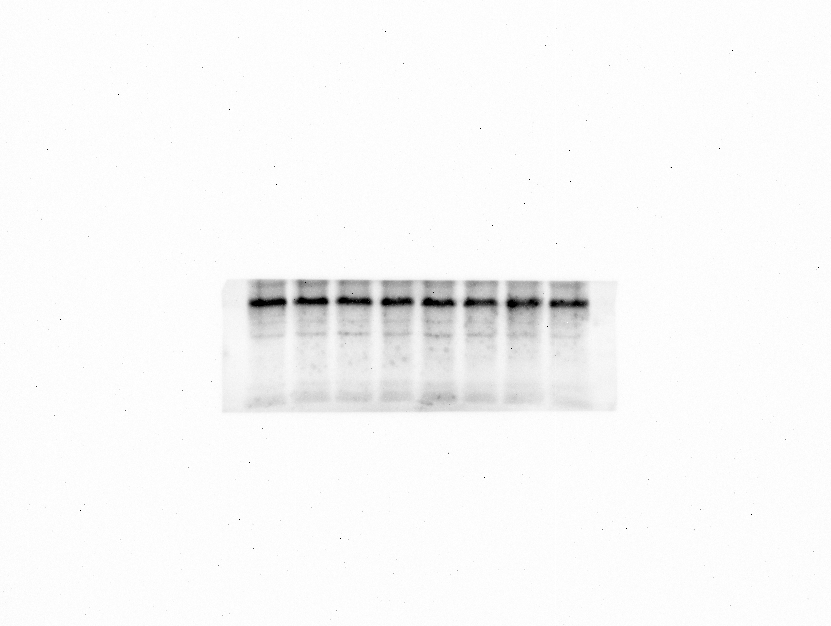

Supplement: Supplementary file 3 [file DataSheet1.ZIP › Original Images for Blots/WB-lung/P-MYPT1 and MYPT1/MYPT1/GAPDH-2.png]

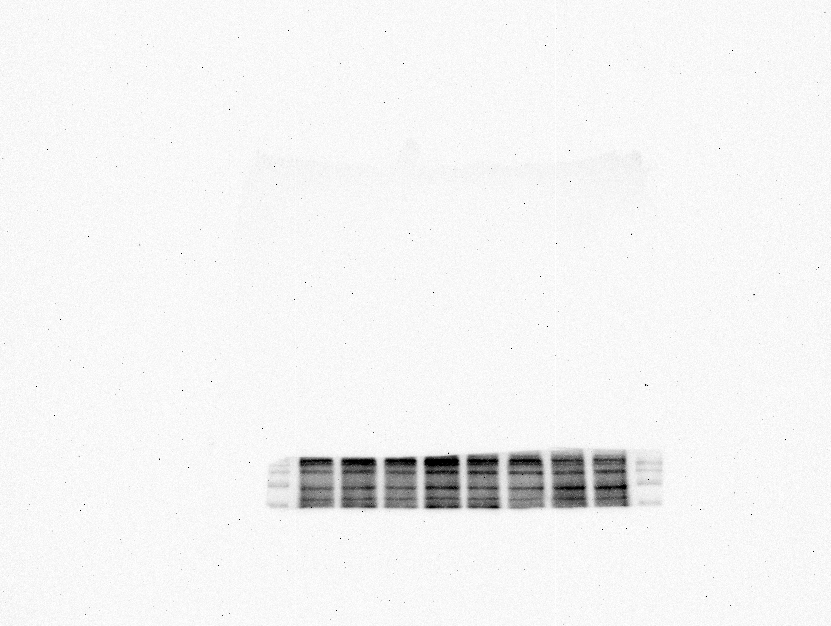

Supplement: Supplementary file 3 [file DataSheet1.ZIP › Original Images for Blots/WB-lung/P-MYPT1 and MYPT1/MYPT1/MYPT1-1.png]

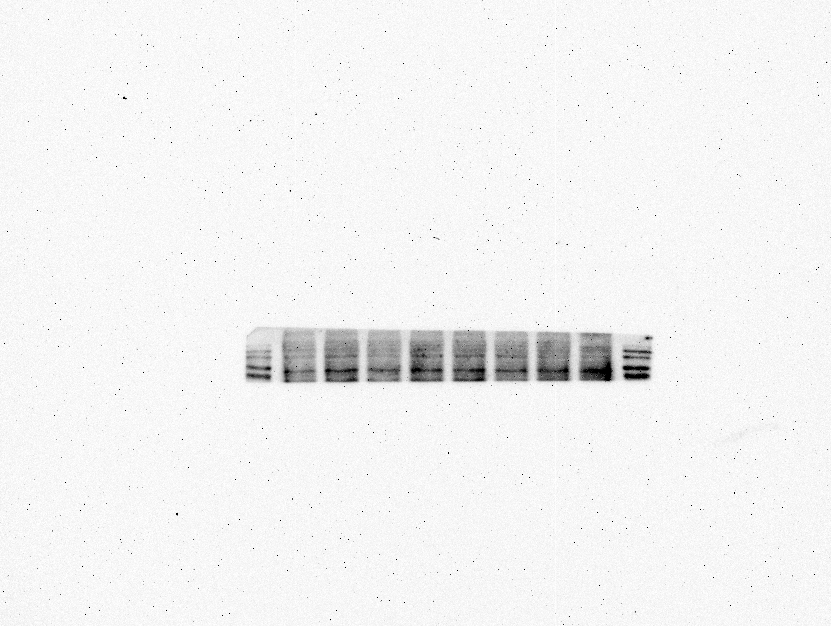

Supplement: Supplementary file 3 [file DataSheet1.ZIP › Original Images for Blots/WB-lung/P-MYPT1 and MYPT1/MYPT1/MYPT1-2.png]

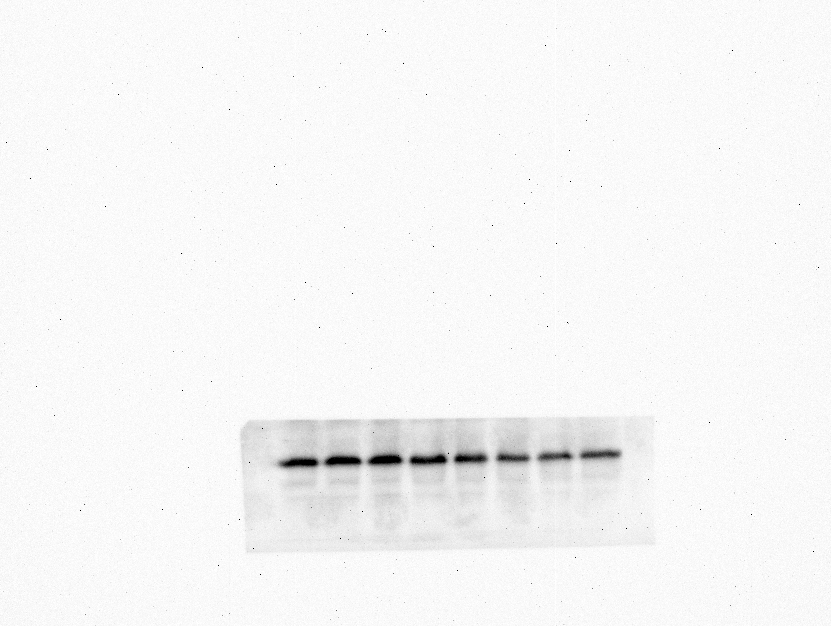

Supplement: Supplementary file 3 [file DataSheet1.ZIP › Original Images for Blots/WB-lung/P-MYPT1 and MYPT1/P-MYPT1/GAPDH-2.png]

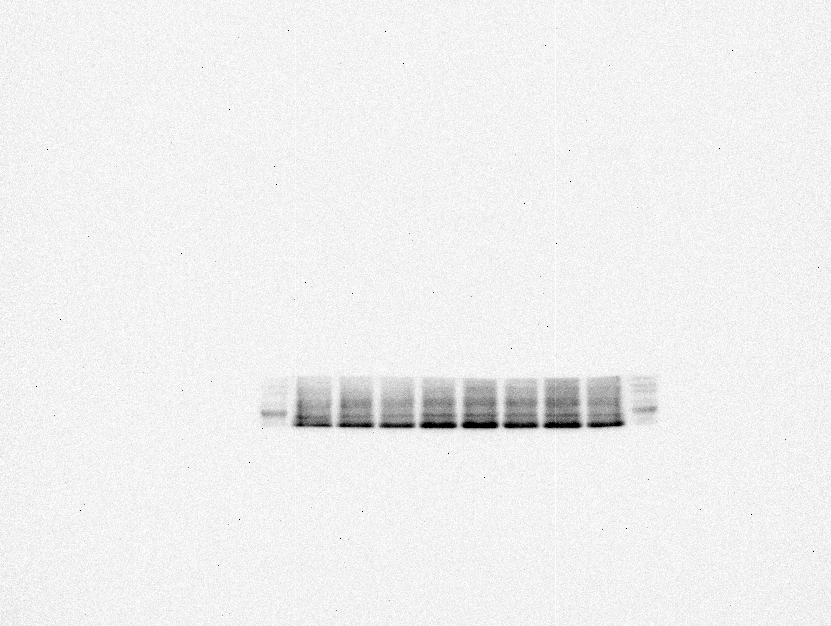

Supplement: Supplementary file 3 [file DataSheet1.ZIP › Original Images for Blots/WB-lung/P-MYPT1 and MYPT1/P-MYPT1/P-MYPT1-1.png]

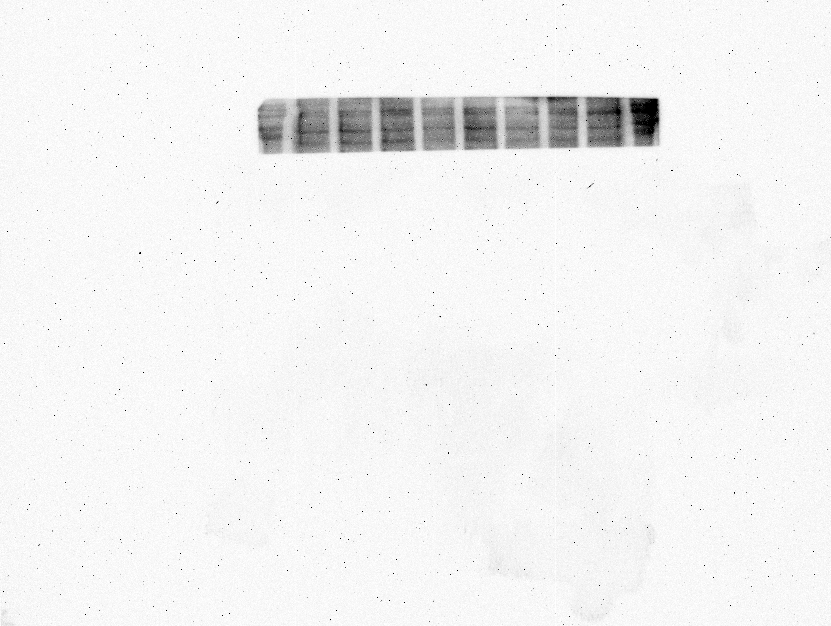

Supplement: Supplementary file 3 [file DataSheet1.ZIP › Original Images for Blots/WB-lung/P-MYPT1 and MYPT1/P-MYPT1/P-MYPT1-2.png]
